# Combined noninvasive brain stimulation and acupuncture for post-stroke motor recovery: a systematic review and network meta-analysis

**DOI:** 10.3389/fneur.2026.1901205

**Published:** 2026-08-20

**Authors:** Qingping Su, Changmei Lu, Yuping Yang, Bao Wu, Jingfang Zhu, Minguang Yang, Ying Xu, Zhizhen Liu, Jing Tao

**Affiliations:** 1 College of Rehabilitation Medicine, Fujian University of Traditional Chinese Medicine, Fuzhou, Fujian, China; 2 Rehabilitation Medicine Center, Fuzhou University Affiliated Provincial Hospital, Fuzhou, Fujian, China; 3 National-Local Joint Engineering Research Center of Rehabilitation Medicine Technology, Fujian University of Traditional Chinese Medicine, Fuzhou, Fujian, China; 4 Traditional Chinese Medicine Rehabilitation Research Center of State Administration of Traditional Chinese Medicine, Fujian University of Traditional Chinese Medicine, Fuzhou, Fujian, China; 5 Laboratory of Orthopedics & Traumatology of Traditional Chinese Medicine and Rehabilitation (Fujian University of Traditional Chinese Medicine), Ministry of Education, Fuzhou, Fujian, China

**Keywords:** acupuncture, motor recovery, network meta-analysis, noninvasive brain stimulation, stroke, systematic review

## Abstract

**Objective:**

Stroke is highly prevalent among older adults, and age-related reductions in neuroplastic reserve may limit motor recovery. This systematic review and network meta-analysis aimed to compare and rank the effects of noninvasive brain stimulation combined with acupuncture on post-stroke motor recovery, spasticity, balance, and activities of daily living (ADL).

**Methods:**

PubMed, Embase, the Cochrane Library, Web of Science, Scopus, CNKI, SinoMed, Wanfang, and CQVIP were searched from inception to July 1, 2026. Randomized controlled trials evaluating noninvasive brain stimulation, including TMS-based NIBS or transcranial direct current stimulation, combined with acupuncture or electroacupuncture were included. Pairwise and frequentist random-effects network meta-analyses were performed. Risk of bias was assessed using the Cochrane tool, and confidence in network estimates was evaluated using CINeMA.

**Results:**

Forty-six randomized controlled trials involving 4,080 participants were included. Combined interventions generally showed greater effects than single-modality therapies or conventional rehabilitation, although heterogeneity was substantial. In the network meta-analysis, both TMS-based NIBS plus acupuncture and tDCS plus acupuncture showed favorable ranking probabilities across several outcomes. tDCS plus acupuncture ranked highly for FMA total, FMA-UE, and ADL, whereas TMS-based NIBS plus acupuncture ranked favorably for FMA-LE, spasticity, and balance. Differences between the two combined regimens were generally not statistically significant. CINeMA indicated moderate, low, or very low confidence across network estimates, with no high-confidence evidence.

**Conclusion:**

Combined noninvasive brain stimulation and acupuncture may provide additional benefits for post-stroke motor and functional recovery. However, current findings should be interpreted cautiously because of substantial heterogeneity, sparse evidence for some intervention nodes, risk-of-bias concerns, and limited confidence in several network estimates.

**Systematic review registration:**

https://www.crd.york.ac.uk/PROSPERO/view/CRD420251051398, Identifier: CRD420251051398.

## 1 Introduction

Stroke remains one of the leading causes of death and long-term disability worldwide and imposes a substantial burden on patients, families, and health-care systems (1). Motor impairment is among the most common and disabling sequelae after stroke, limiting independence in activities of daily living and reducing quality of life (2). Although spontaneous recovery and rehabilitation-induced gains may occur, a considerable proportion of stroke survivors continue to experience persistent upper limb dysfunction, lower limb impairment, spasticity, balance deficits, and reduced functional independence (3). Therefore, identifying interventions that can enhance post-stroke motor recovery and functional restoration remains a central priority in neurorehabilitation.

Importantly, stroke is closely associated with aging, and many stroke survivors are older adults (4). Age-related changes in the central nervous system, including reduced corticospinal excitability, impaired synaptic plasticity, altered interhemispheric balance, diminished sensorimotor integration, and less efficient network reorganization, may constrain the capacity for motor recovery after stroke (5, 6). From the perspective of aging neuroscience, post-stroke rehabilitation is therefore not only a problem of peripheral motor restoration but also a challenge of promoting adaptive neuroplasticity in an aging or aging-vulnerable brain (7). Interventions capable of modulating cortical excitability, enhancing sensorimotor integration, and facilitating functional network reorganization may be particularly relevant for improving rehabilitation outcomes in this population.

Noninvasive brain stimulation (NIBS), including transcranial magnetic stimulation-based protocols such as repetitive transcranial magnetic stimulation (rTMS) and theta-burst stimulation, as well as transcranial direct current stimulation (tDCS), has attracted increasing attention as a neuroplasticity-oriented strategy for post-stroke rehabilitation (8, 9). These techniques can modulate cortical excitability, rebalance abnormal interhemispheric inhibition, and facilitate activity-dependent reorganization of motor networks (5). However, the clinical effects of NIBS alone are variable across patients, partly because recovery after stroke is influenced by lesion characteristics, disease stage, stimulation parameters, baseline motor impairment, and the individual capacity for neuroplastic adaptation. Thus, NIBS may need to be integrated with complementary peripheral or task-oriented interventions to maximize its therapeutic potential.

Acupuncture, including electroacupuncture, remains widely used in traditional rehabilitation practice. Evidence suggests that it can improve limb motor function (10), relieve spasticity (11), and enhance balance (12). Although the mechanisms are still being clarified, acupuncture is increasingly discussed within modern frameworks of motor control and neural plasticity. From a mechanistic perspective, NIBS and acupuncture may influence complementary levels of the motor system. NIBS may prime central motor circuits through top-down modulation, whereas acupuncture may refine afferent input and sensorimotor integration through bottom-up pathways. Combining these interventions may therefore provide a dual neuromodulatory strategy to enhance motor learning, muscle tone regulation, postural control, and functional task performance after stroke.

Several clinical trials have investigated combined protocols involving rTMS or tDCS with acupuncture for post-stroke motor recovery (13, 14). Previous evidence syntheses have suggested that rTMS combined with acupuncture may improve upper limb motor dysfunction after stroke (15, 16). However, existing reviews have generally focused on rTMS plus acupuncture, relied mainly on traditional pairwise meta-analysis, and emphasized upper limb outcomes. As a result, the comparative effectiveness of different combined neuromodulatory regimens remains unclear. In particular, it is still uncertain whether rTMS-, tDCS-, or iTBS-based combinations differ in their relative effects on multiple clinically relevant outcomes, including overall motor function, upper and lower limb recovery, spasticity, balance, and activities of daily living.

To address these gaps, we conducted a systematic review and network meta-analysis of randomized controlled trials evaluating NIBS combined with acupuncture or electroacupuncture for post-stroke rehabilitation. Unlike previous pairwise meta-analyses focusing mainly on rTMS plus acupuncture or upper limb recovery, this study integrated direct and indirect evidence across multiple combined neuromodulatory strategies, including TMS-based and tDCS-based regimens combined with acupuncture or electroacupuncture. We aimed to compare and rank the effects of these interventions on motor function, muscle tone, balance, and activities of daily living, thereby providing a more comprehensive evidence base for selecting neuroplasticity-oriented rehabilitation strategies after stroke. By framing combined NIBS and acupuncture as a central–peripheral neuromodulatory approach, this review also seeks to inform future research on aging-related neurorehabilitation and post-stroke network recovery.

## 2 Methods

### 2.1 Protocol registration and reporting standards

We developed a protocol for this systematic review in advance and registered it in the International Prospective Register of Systematic Reviews (PROSPERO) on 13 May 2025 (registration number CRD420251051398). The protocol was prepared in accordance with the Preferred Reporting Items for Systematic Reviews and Meta-Analyses Protocols (PRISMA-P) statement (17, 18). The conduct and reporting of this review followed the Preferred Reporting Items for Systematic Reviews and Meta-Analyses (PRISMA) statement (19) and its extension for network meta-analysis (NMA) (20) and adhered to the methodological standards recommended by the Cochrane Collaboration (21). The completed PRISMA-NMA checklist is provided as Supplementary Table S1. The aim of this study was to synthesize evidence on the effects of NIBS combined with acupuncture on motor recovery after stroke, to provide an evidence-based reference for clinical practice and to identify gaps in the current literature.

### 2.2 Eligibility criteria

Studies were considered eligible if they met all the following criteria: (1) participants were adult patients with a clinical diagnosis of stroke who were in the rehabilitation phase; (2) the study design was a parallel-group randomized controlled trial (RCT); (3) the experimental intervention consisted of NIBS, such as rTMS or tDCS, combined with acupuncture, including electroacupuncture; (4) control groups received conventional rehabilitation, acupuncture alone, NIBS alone, sham stimulation, or sham acupuncture, as appropriate; and (5) at least one of the prespecified outcomes was reported. For the network meta-analysis, intervention nodes were defined according to the active NIBS modality and whether acupuncture or electroacupuncture was combined. Conventional low- and high-frequency rTMS, intermittent theta-burst stimulation (iTBS), continuous theta-burst stimulation (cTBS), and mixed magnetic stimulation protocols were classified as TMS-based NIBS because all these protocols use transcranial magnetic stimulation as the active stimulation modality. However, theta-burst stimulation differs from conventional rTMS in stimulation pattern and physiological dosing. Therefore, iTBS-, cTBS-, and mixed-protocol studies were identified separately during data extraction. Because the number of these studies was small and insufficient to form stable independent nodes, they were included in the broader TMS-based NIBS node in the primary network meta-analysis. Prespecified outcomes included motor function and muscle tone outcomes, namely the Fugl-Meyer Assessment (FMA), including the upper extremity subscale (FMA-UE), the lower extremity subscale (FMA-LE), and the FMA total score, as well as the Modified Ashworth Scale (MAS) for spasticity. Functional outcomes included the Berg Balance Scale (BBS) for balance and activities of daily living (ADL) measures, specifically the Modified Barthel Index (MBI) or Barthel Index (BI). The exclusion criteria were as follows: (1) nonrandomized studies; (2) reviews, conference abstracts, dissertations, or theses; (3) duplicate publications or studies with overlapping samples; (4) studies for which the full text could not be obtained or for which the necessary data were unavailable for eligibility assessment or quantitative synthesis; and (5) animal studies. Studies were eligible regardless of journal indexing status. Eligibility was determined solely on the basis of the prespecified study design, participants, interventions, comparators, and outcomes.

### 2.3 Information sources and search strategy

We systematically searched nine English and Chinese electronic databases from inception to July 1, 2026: PubMed, Embase, the Cochrane Library, Web of Science, Scopus, China National Knowledge Infrastructure (CNKI), the Chinese Biomedical Literature Database (SinoMed), Wanfang Data, and the VIP Chinese Science and Technology Journal Database (CQVIP). The search strategy was developed according to the PICOS framework and combined controlled vocabulary terms, including MeSH terms and Emtree terms where applicable, with free-text terms related to stroke, noninvasive brain stimulation, acupuncture, motor and functional outcomes, and randomized controlled trials.

To improve the comprehensiveness of the search, especially for tDCS- and theta-burst stimulation-based combined interventions, we also manually searched the reference lists of included studies and relevant reviews. The full search strategies for all databases, including controlled vocabulary terms, free-text terms, search logic, and the number of records retrieved, are provided in Supplementary Tables S2, S3.

### 2.4 Study selection

Two reviewers (Qingping Su and Yuping Yang) independently managed and screened all the retrieved records via EndNote X9 software, following the predefined inclusion and exclusion criteria. First, titles and abstracts were screened to exclude clearly irrelevant records. Full texts were then obtained for studies that were potentially eligible, and their eligibility was assessed in detail. Any disagreements during the selection process were resolved through discussion or, when necessary, by consulting a third reviewer (Changmei Lu).

The study selection process followed the PRISMA flow diagram, which is presented in the Results section, including the number of records identified at each stage, the numbers excluded, and the specific reasons for exclusion at the full-text stage.

### 2.5 Data collection and management

Two reviewers (Qingping Su and Yuping Yang) independently extracted data from the included studies via a predesigned standardized data extraction form. The following information was collected. (1) General study characteristics, including first author, year of publication, country, and study design. (2) Participant characteristics, including sample size, sex distribution, age, stroke type, and time since stroke onset. (3) Details of the interventions, including the type and parameters of the NIBS, the acupuncture protocol, and the mode of combined application. (4) Outcome measures, including baseline and post treatment means and standard deviations for all primary and secondary outcomes. (5) Methodological information, including methods of randomization, allocation concealment, blinding procedures, and other design features. We additionally extracted language of publication, country or region, acupuncture type, NIBS node classification, trial registration or protocol availability, analysis population (intention-to-treat or per-protocol), dropout information, treatment compliance, and adverse events when reported.

When data were missing or ambiguously reported, we contacted the corresponding authors by email to obtain the original data. If no response was received, we attempted to estimate values via digital extraction from figures or via coefficients reported in the article. Any discrepancies in data collection were resolved through discussion or by consulting a third reviewer. All the extracted data were cross-checked by a third researcher (Bao Wu) to ensure accuracy.

### 2.6 Risk of bias assessment

The methodological quality and risk of bias of the included RCTs were assessed via the Cochrane Collaboration risk of bias tool (RoB 1.0). This tool covers seven domains: random sequence generation, allocation concealment, blinding of participants and personnel, blinding of outcome assessment, incomplete outcome data, selective outcome reporting, and other sources of bias. Each domain was judged as having a low, unclear, or high risk of bias.

We did not exclude studies solely on the basis of a high risk of bias in individual domains or overall risk. Instead, Risk-of-bias judgments were considered when interpreting the findings and were incorporated into the CINeMA assessment of within-study bias. Two independent reviewers (Qingping Su and Yuping Yang) conducted the risk of bias assessment via Review Manager 5.4 (RevMan 5.4). Any disagreements were resolved through discussion or by consultation with a third reviewer (Changmei Lu). The results of the risk of bias assessment are presented as a risk of bias summary and a risk of bias graph generated by RevMan 5.4.

To improve interpretability of the risk-of-bias assessment, domain-specific judgments were made according to predefined criteria. Studies were judged as low risk for random sequence generation when an appropriate randomization method, such as a random number table or computer-generated sequence, was clearly reported; unclear risk when only “random allocation” was mentioned without further details; and high risk when quasi-random methods were used. Allocation concealment and blinding domains were judged according to whether adequate concealment methods, credible sham procedures, or blinded outcome assessment were explicitly reported. Selective reporting was assessed by considering the availability of trial registration or protocols and whether prespecified outcomes appeared to be completely reported.

### 2.7 Data synthesis and statistical analysis

All the statistical analyses were performed using Stata version 18.0 (Stata Corp LLC, College Station, TX, USA). Analyses, including pairwise meta-analyses and NMA, were conducted within a frequentist random-effects framework.

For pairwise meta-analyses, we conducted head-to-head comparisons for each outcome, including FMA-UE, FMA-LE, FMA total score, MAS, BBS, and ADL. ADL was measured using the Barthel Index (BI) or Modified Barthel Index (MBI). BI and MBI were pooled as the primary ADL outcome because both are widely used 0–100-point measures of independence in activities of daily living after stroke and have comparable clinical interpretations. To examine whether this pooling decision influenced the ADL findings, sensitivity analyses were conducted by separately analyzing BI-only and MBI-only studies. For continuous outcomes measured using the same or comparable scales, mean differences (MDs) with 95% confidence intervals (CIs) were calculated. Statistical heterogeneity was assessed using the I^2^ statistic and Cochran’s Q test. An I^2^ value greater than 50% was considered to indicate substantial heterogeneity. Random-effects models were used because clinical and methodological heterogeneity was expected across trials. When sufficient studies were available, subgroup analyses were conducted according to intervention comparisons to explore potential sources of heterogeneity.

For the network meta-analysis, network plots were first constructed to visualize the pattern of direct and indirect comparisons among interventions. A frequentist random-effects consistency model was used as the primary analytic approach. For each comparison, MDs and 95% CIs were estimated and summarized in league tables. Interventions were ranked using surface under the cumulative ranking curve (SUCRA) values and cumulative ranking plots, and SUCRA values were used to describe the probability of each intervention being ranked higher within the network. SUCRA rankings were interpreted as relative ranking probabilities rather than definitive evidence of superior therapeutic efficacy. Local inconsistency was considered descriptively based on network structure and CINeMA incoherence judgments. Comparison-adjusted funnel plots were generated to evaluate potential publication bias and small-study effects. Theta-burst and mixed magnetic stimulation studies were identified separately, and their potential influence was considered descriptively because the number of such studies was too small to support stable independent network analyses. The transitivity assumption was assessed by comparing key potential effect modifiers across treatment comparisons, including age, stroke type, disease duration, baseline motor impairment, NIBS modality and parameters, acupuncture type, treatment frequency, and treatment duration.

### 2.8 Confidence in network estimates

The confidence in network estimates was assessed using CINeMA (Confidence in Network Meta-Analysis), a GRADE-based framework specifically developed for network meta-analysis. CINeMA evaluates six domains: within-study bias, reporting bias, indirectness, imprecision, heterogeneity, and incoherence. The risk-of-bias judgments from the Cochrane RoB 1.0 tool were used to inform the within-study bias domain. For each outcome, clinically relevant comparisons were assessed, including combined regimens versus single-modality therapies or conventional rehabilitation, and head-to-head comparisons between combined regimens when available. Judgments for each domain were classified as no concerns, some concerns, or major concerns, and the overall confidence in each network estimate was rated as high, moderate, low, or very low. Detailed CINeMA judgments and reasons for downgrading are provided in Supplementary Table S5.

## 3 Results

### 3.1 Study selection

**Figure 1:**
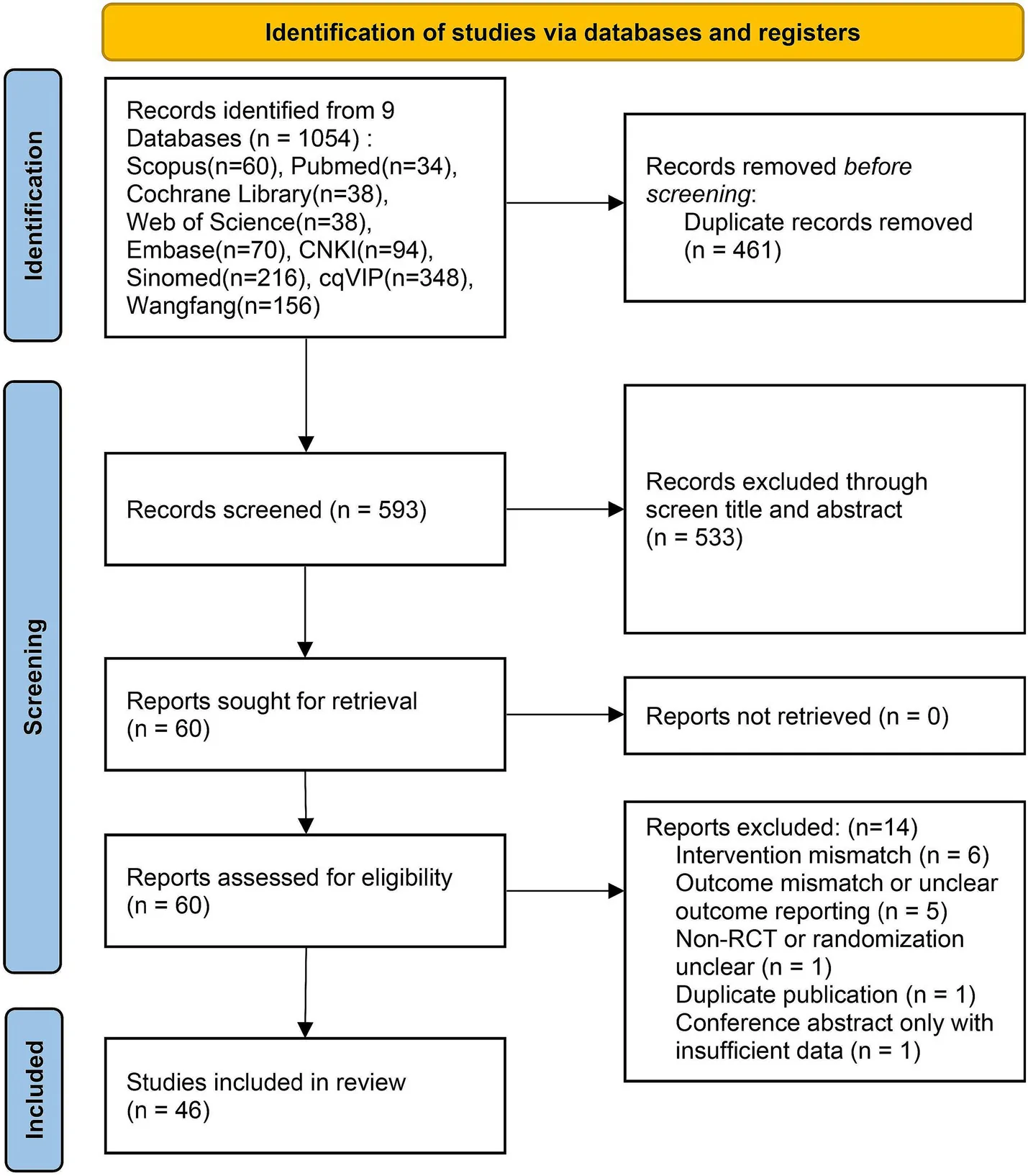
PRISMA flow diagram of study selection.

A total of 1,054 records were identified through systematic searches of nine databases (Scopus, PubMed, the Cochrane Library, Web of Science, Embase, CNKI, SinoMed, CQVIP, and Wanfang). After removing 461 duplicates, 593 records remained for title and abstract screening. At this stage, 533 records were excluded. Sixty full-text reports were retrieved and assessed for eligibility. No reports were unavailable for full-text assessment. Fourteen reports were excluded for the following reasons: intervention mismatch (*n* = 6), outcome mismatch or unclear outcome reporting (*n* = 5), non-RCT design or unclear randomization (*n* = 1), duplicate publication (n = 1), and conference abstract only with insufficient data (*n* = 1). Finally, 46 randomized controlled trials were included in the systematic review and network meta-analysis (Figure 1). A detailed list of full-text excluded studies and reasons for exclusion is provided in Supplementary Table S4.

### 3.2 Characteristics of the included studies

In total, 46 RCTs involving 4,080 participants were included (Supplementary Table S5). The sample size per trial ranged from 17 to 164. All studies enrolled adult patients after stroke, with mean ages ranging approximately from 55 to 73 years. Both ischemic and hemorrhagic stroke were represented, with ischemic stroke being more common overall. Disease duration varied across studies and covered the acute, subacute, and chronic phases, although most trials focused on patients in the subacute rehabilitation phase. Most included trials were conducted in China and published in Chinese, with only a small number of studies conducted in Korea or published in English. This regional and language concentration was considered when interpreting the generalizability of acupuncture-related findings.

The experimental interventions mainly involved NIBS combined with acupuncture or electroacupuncture. rTMS-based regimens were the most frequently investigated, followed by tDCS-based regimens. A small number of studies used iTBS or mixed rTMS/iTBS protocols. Most rTMS protocols applied low-frequency stimulation over the contralesional primary motor cortex, whereas some trials used high-frequency stimulation over the ipsilesional motor cortex, bilateral motor cortices, or the dorsolateral prefrontal cortex. For tDCS, the anode was commonly placed over the ipsilesional motor cortex, with the cathode placed over the contralateral supraorbital region, shoulder, or motor cortex.

Acupuncture interventions included scalp acupuncture, body acupuncture, electroacupuncture, and several named or standardized acupuncture regimens. Control conditions included conventional rehabilitation therapy (CRT), acupuncture alone, NIBS alone, sham stimulation, or sham acupuncture. Treatment was most commonly delivered five sessions per week, with intervention durations ranging from 2 to 6 weeks, although some trials used longer treatment courses or included follow-up assessments.

The main outcomes included FMA total score, FMA-UE, FMA-LE, MAS, BBS, and ADL measured by BI or MBI. Other less frequently reported functional outcomes were extracted when available but were not included as primary nodes in the main quantitative synthesis. Detailed characteristics of the included studies are provided in Supplementary Table S5. The distribution of key clinical and intervention characteristics was generally comparable across major comparisons, although variation remained in disease stage, baseline impairment, NIBS parameters, acupuncture modality, and treatment duration, which were considered when interpreting transitivity and indirectness.

### 3.3 Risk of bias assessment

**Figure 2:**
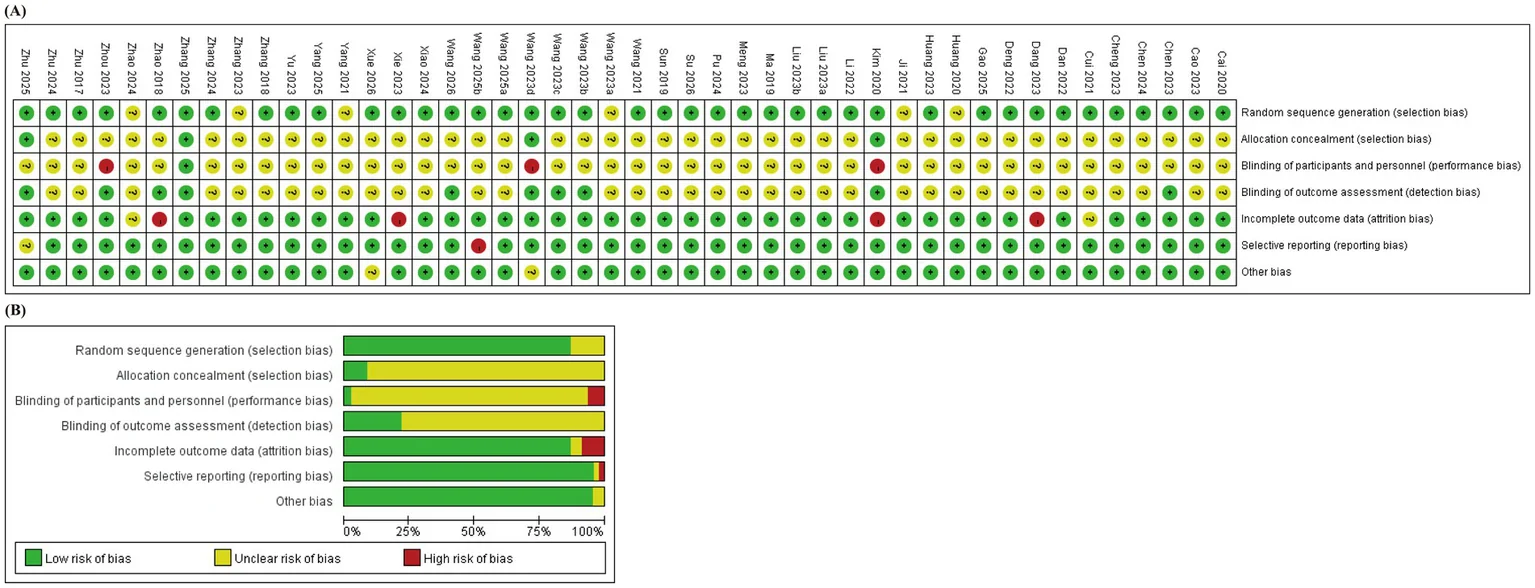
Risk of bias assessment for the included studies. **(A)** Domain-level judgments for individual studies. **(B)** Overall proportions of low, unclear, and high risk of bias across domain.

The risk of bias of the included RCTs was evaluated using the Cochrane RoB 1.0 tool (Figure 2). Overall, the included studies showed some methodological concerns, mainly related to allocation concealment and blinding procedures.

For random sequence generation, most studies reported appropriate randomization methods and were judged as having a low risk of bias, whereas a small proportion provided insufficient details and were rated as unclear risk. In contrast, allocation concealment was poorly reported in most studies and was therefore mainly judged as unclear risk.

For performance bias, most studies did not provide sufficient information on blinding of participants and personnel or on the use and credibility of sham procedures. Therefore, this domain was mainly judged as having an unclear risk of bias, with a small number of studies rated as high risk. For detection bias, only a minority of studies explicitly reported that outcome assessors were blinded to group allocation and were judged as having a low risk of bias. Most studies did not clearly describe assessor blinding and were therefore rated as unclear risk.

With respect to attrition bias, most studies had complete outcome data and were rated as having a low risk of bias, although a small number of studies were judged as high risk because of incomplete outcome data. Selective reporting and other bias were generally judged as low risk, although trial registration and prespecified protocols were not consistently reported. Taken together, the main methodological limitations were insufficient reporting of allocation concealment and blinding procedures, together with a small number of studies at high risk for performance or attrition bias. These risk-of-bias judgments were considered when interpreting the findings and were incorporated into the CINeMA assessment of within-study bias.

### 3.4 Results of pairwise meta-analyses

Pairwise meta-analyses were performed for FMA total, FMA-UE, FMA-LE, and ADL outcomes (Supplementary Figures S1–S4). Overall, combined NIBS and acupuncture showed significantly greater improvements than single-modality interventions or conventional rehabilitation across these outcomes. However, substantial heterogeneity was observed, and therefore random-effects models were used. To explore potential sources of heterogeneity, subgroup analyses were conducted according to intervention comparisons.

For FMA total score, combined interventions showed significant improvement compared with control interventions (MD = 9.14, 95% CI [6.82 to 11.47], *p* < 0.001), although substantial heterogeneity was observed (*I*^2^ = 95.3%). Subgroup analysis showed that TMS-based NIBS plus acupuncture significantly improved FMA compared with TMS alone (MD = 7.75, 95% CI [5.39 to 10.12], *p* < 0.001) and conventional rehabilitation therapy (MD = 7.49, 95% CI [1.06 to 13.92], *p* = 0.022). However, the difference between TMS-based NIBS plus acupuncture and acupuncture alone was not statistically significant (MD = 6.33, 95% CI [−0.40 to 13.06], *p* = 0.065). In addition, tDCS plus acupuncture showed significant benefit compared with tDCS alone (MD = 24.23, 95% CI [21.37 to 27.08], *p* < 0.001).

For FMA-UE, combined treatment significantly improved upper limb motor function overall (MD = 6.25, 95% CI [5.16 to 7.34], *p* < 0.001), with substantial heterogeneity (*I*^2^ = 87.2%). All analyzed subgroups favored combined treatment, including TMS-based NIBS plus acupuncture versus TMS-based NIBS alone, acupuncture alone, or CRT, and tDCS plus acupuncture versus acupuncture alone or tDCS alone. No significant subgroup difference was detected (*p* = 0.549), indicating that the beneficial effect on upper limb motor function was generally consistent across different comparator types.

For FMA-LE, combined treatment significantly improved lower limb motor function overall (MD = 4.84, 95% CI [3.66 to 6.01], *p* < 0.001), with high heterogeneity (*I*^2^ = 85.2%). TMS-based NIBS plus acupuncture was significantly superior to TMS-based NIBS alone and CRT, whereas the comparison with acupuncture alone did not reach statistical significance. No significant subgroup difference was observed (*p* = 0.904), suggesting that the effect on lower limb motor recovery was relatively consistent across available comparisons.

For ADL measured by BI or MBI, combined treatment showed a significant overall benefit (MD = 8.85, 95% CI [7.69 to 10.02]), with high heterogeneity (*I*^2^ = 89.6%). Subgroup analysis showed significant benefits for TMS-based NIBS plus acupuncture compared with TMS-based NIBS alone, acupuncture alone, or CRT, and for tDCS plus acupuncture compared with acupuncture alone or tDCS alone. Significant subgroup differences were detected (*p* = 0.007), suggesting that the magnitude of ADL improvement may vary according to the type of NIBS modality and comparator.

Overall, the pairwise meta-analyses supported the potential benefit of combined NIBS and acupuncture for motor and functional recovery after stroke. Nevertheless, because heterogeneity remained substantial across several outcomes and some subgroup comparisons were based on a limited number of studies, these findings should be interpreted descriptively and cautiously.

### 3.5 Results of the network meta-analysis

#### 3.5.1 Network geometry

Network plots were generated for each outcome to illustrate the pattern of direct and indirect comparisons among interventions. In these plots, nodes represent interventions, and their size is proportional to the total sample size for that intervention, whereas edges represent head–to–head comparisons between interventions, and their thickness reflects the number of trials informing each comparison.

**Figure 3:**
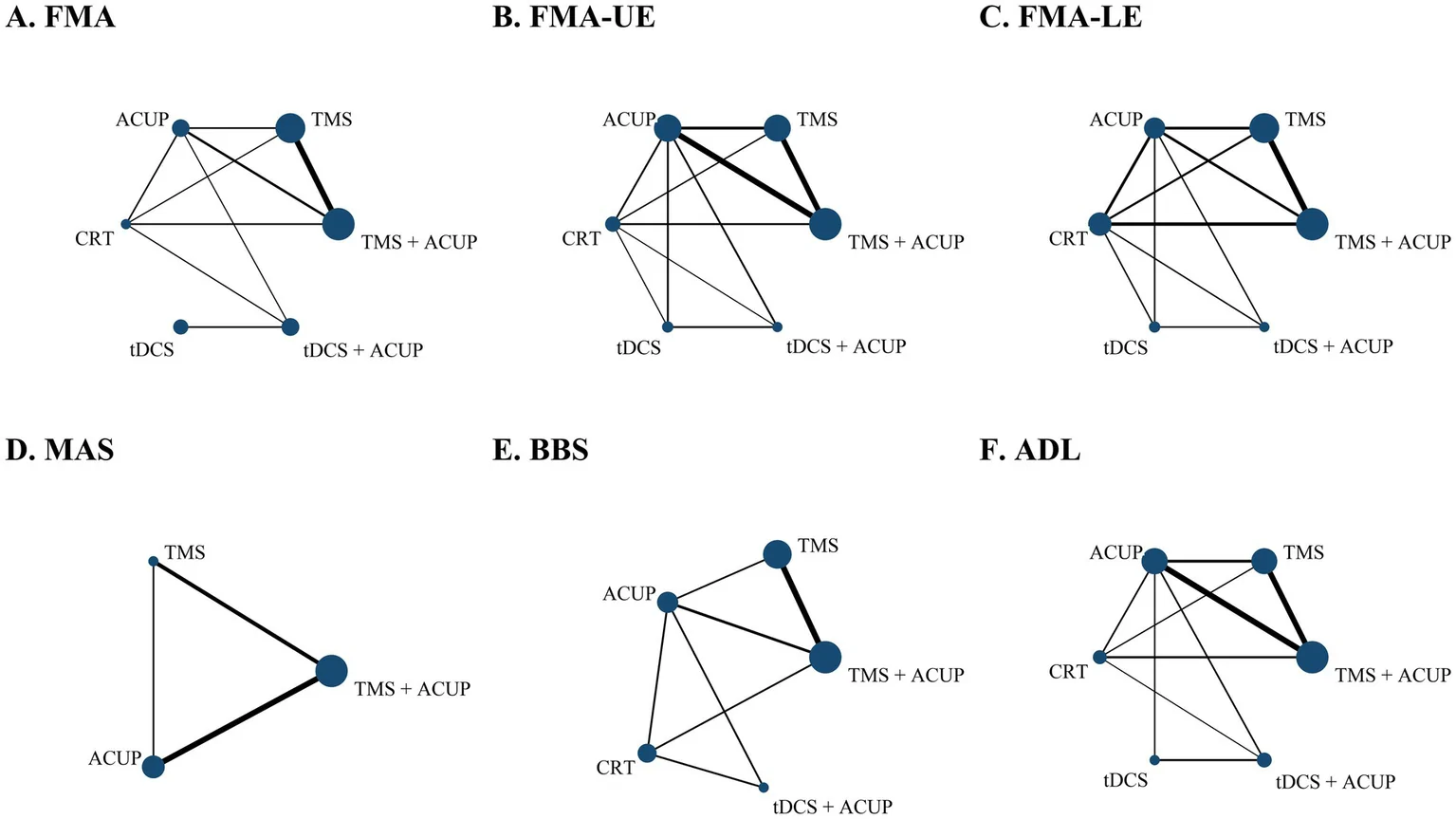
Network plots for each outcome: **(A)** FMA; **(B)** FMA-UE; **(C)** FMA-LE; **(D)** MAS; **(E)** BBS; **(F)** ADL. TMS denotes TMS-based NIBS, including conventional rTMS and sparse theta-burst or mixed magnetic stimulation protocols.

Across all outcomes, TMS-based NIBS plus acupuncture, TMS-based NIBS alone, and acupuncture alone contributed the largest total sample sizes. The most frequent direct comparisons were TMS-based NIBS plus acupuncture versus acupuncture alone and TMS-based NIBS plus acupuncture versus TMS-based NIBS alone. The TMS-based nodes were driven mainly by conventional rTMS studies, whereas theta-burst or mixed magnetic stimulation protocols contributed only a small proportion of the evidence. The network structures for FMA total, FMA-UE, FMA-LE, MAS, BBS, and ADL are shown in Figure 3.

#### 3.5.2 Motor function

##### 3.5.2.1 FMA

Nineteen trials reporting FMA total scores and involving six interventions were included in the network meta-analysis (22–40). As shown in Table 1, TMS-based NIBS combined with acupuncture produced significantly greater improvements in overall motor function than TMS-based NIBS alone (MD = 8.07, 95% CI [4.47 to 11.67]), acupuncture alone (MD = 5.48, 95% CI [0.86 to 11.82]), CRT (MD = 13.67, 95% CI [5.61 to 21.72]), and tDCS alone (MD = 22.09, 95% CI [6.70 to 37.49]).

**Table 1:** Network meta-analysis results for FMA total [MD (95% CI)].

TMS + ACUP					
8.07 (4.47,11.67)^a^	TMS				
5.48 (0.86,11.82) ^a^	−2.59 (−9.45,4.27)	ACUP			
13.67 (5.61,21.72)^a^	5.60 (−2.94,14.14)	8.19 (0.02,16.36)^a^	CRT		
22.09 (6.70,37.49)^a^	14.03 (−1.61,29.66)	16.61 (1.85,31.38)^a^	8.43 (−6.33,23.19)	tDCS	
−2.21 (−14.53,10.10)	−10.28 (−22.90,2.33)	−7.70 (−19.21,3.82)	−15.88 (−27.39,–4.37)^a^	−24.31 (−33.55,–15.07)^a^	tDCS + ACUP

^a^Indicates a statistically significant comparison, defined as a 95% CI that does not include 0.

tDCS combined with acupuncture was significantly more effective than CRT (MD = −15.88, 95% CI [−27.39 to −4.37]) and tDCS alone (MD = −24.31, 95% CI [−33.55 to −15.07]). The differences between tDCS plus acupuncture and TMS-based NIBS plus acupuncture was not statistically significant (MD = −2.21, 95% CI [−14.53 to 10.10]).

**Figure 4:**
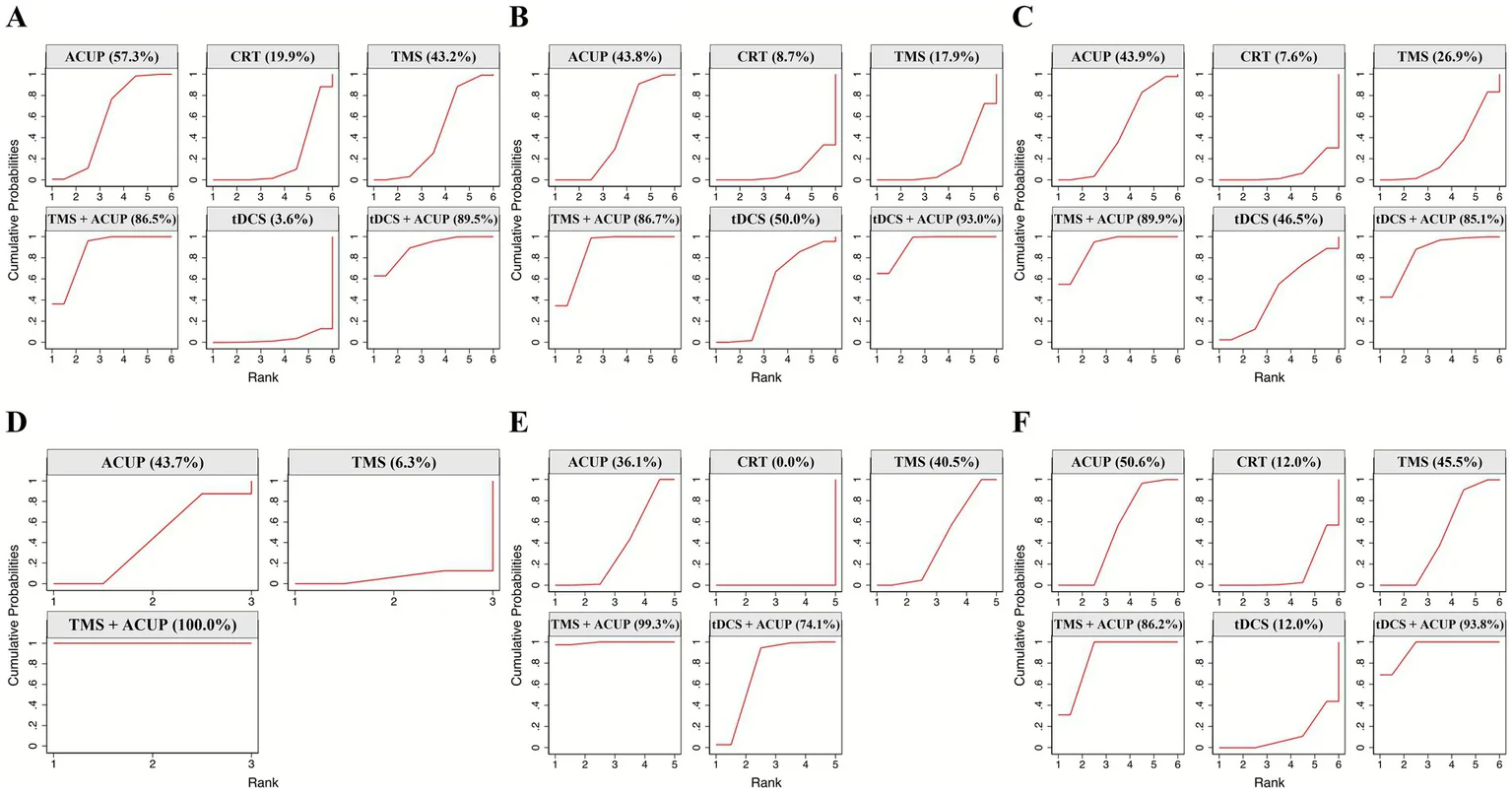
SUCRA plots for each outcome: **(A)** FMA; **(B)** FMA-UE; **(C)** FMA-LE; **(D)** MAS; **(E)** BBS; **(F)** ADL.

According to SUCRA values (Figure 4A), the ranking probabilities for FMA total were as follows: tDCS plus acupuncture (89.5%), TMS-based NIBS plus acupuncture (86.5%), acupuncture alone (57.3%), TMS-based NIBS alone (43.2%), CRT (19.9%), and tDCS alone (3.6%). These findings suggest that both tDCS plus acupuncture and TMS-based NIBS plus acupuncture had favorable ranking probabilities for improving overall motor function, but their relative difference was not statistically significant and should be interpreted cautiously.

##### 3.5.2.2 FMA-UE

Twenty-seventh trials reported FMA-UE scores and involved six interventions (16, 24, 25, 41–62). The network meta-analysis (Table 2) revealed that TMS-based NIBS combined with acupuncture significantly improved upper limb motor function compared with TMS-based NIBS alone (MD = 7.27, 95% CI [5.56 to 8.99]), acupuncture alone (MD = 5.70, 95% CI [3.96 to 7.43]), CRT (MD = 8.15, 95% CI [5.00 to 11.31]), and tDCS alone (MD = 4.76, 95% CI [0.53 to 9.00]).

**Table 2:** Network meta-analysis results for FMA-UE [MD (95% CI)].

TMS + ACUP					
7.27 (5.56,8.99)^a^	TMS				
5.70 (3.96,7.43)^a^	−1.58 (−3.69,0.54)	ACUP			
8.15 (5.00,11.31)^a^	0.88 (−2.50,4.26)	2.46 (−0.72,5.63)	CRT		
4.76 (0.53,9.00)^a^	−2.51 (−6.92,1.90)	−0.93 (−4.90,3.03)	−3.39 (−7.93,1.15)	tDCS	
−0.86 (−5.13,3.41)	−8.13 (−12.57,–3.70)^a^	−6.56 (−10.56,–2.55)^a^	−9.01 (−13.57,–4.46)^a^	−5.62 (−9.80,–1.45)^a^	tDCS + ACUP

^a^Indicates a statistically significant comparison, defined as a 95% CI that does not include 0.

tDCS combined with acupuncture also showed significantly greater efficacy than TMS-based NIBS alone (MD = −8.13, 95% CI [−12.57 to −3.70]), acupuncture alone (MD = −6.56, 95% CI [−10.56 to −2.55]), CRT (MD = −9.01, 95% CI [−13.57 to −4.46]), and tDCS alone (MD = −5.62, 95% CI [−9.80 to −1.45]). The difference between TMS-based NIBS plus acupuncture and tDCS plus acupuncture was not statistically significant (MD = −0.86, 95% CI [−5.13 to 3.41]).

According to SUCRA values (Figure 4B), tDCS plus acupuncture and TMS-based NIBS plus acupuncture ranked highest for FMA-UE, with ranking probabilities of 93.0 and 86.7%, respectively, followed by tDCS alone (50.0%), acupuncture alone (43.8%), TMS-based NIBS alone (17.9%), and CRT (8.7%). These findings suggest that both tDCS plus acupuncture and TMS-based NIBS plus acupuncture may be associated with greater upper limb motor improvement, but their relative difference was not statistically significant and should be interpreted cautiously.

##### 3.5.2.3 FMA-LE

Eleventh trials reported FMA-LE scores and involved six interventions (24, 25, 38, 44, 47, 49, 51, 61, 63–65). The network meta-analysis (Table 3) indicated that TMS-based NIBS combined with acupuncture significantly improved lower limb motor function compared with TMS-based NIBS alone (MD = 5.01, 95% CI [3.31 to 6.72]), acupuncture alone (MD = 4.02, 95% CI [1.53 to 6.51]), CRT (MD = 6.16, 95% CI [3.80 to 8.51]). However, the differences between TMS-based NIBS plus acupuncture and tDCS alone (MD = 3.59, 95% CI [−1.05 to 8.22]) or tDCS plus acupuncture (MD = 0.37, 95% CI [−4.27 to 5.00]) were not statistically significant.

**Table 3:** Network meta-analysis results for FMA-LE [MD (95% CI)].

TMS + ACUP					
5.01 (3.31,6.72)^a^	TMS				
4.02 (1.53,6.51)^a^	−0.99 (−3.59,1.60)	ACUP			
6.16 (3.80,8.51)^a^	1.14 (−1.44,3.73)	2.13 (−0.45,4.72)	CRT		
3.59 (−1.05,8.22)	−1.43 (−6.15,3.30)	−0.43 (−4.79,3.92)	−2.57 (−6.93,1.79)	tDCS	
0.37 (−4.27,5.00)	−4.65 (−9.37,0.08)	−3.65 (−8.01,0.70)	−5.79 (−10.15,–1.43)^a^	−3.22 (−8.01,1.57)	tDCS + ACUP

^a^Indicates a statistically significant comparison, defined as a 95% CI that does not include 0.

tDCS combined with acupuncture was significantly more effective than CRT (MD = −5.79, 95% CI [−10.15 to −1.43]), but not significantly different from TMS-based NIBS alone, acupuncture alone, or tDCS alone. According to SUCRA values (Figure 4C), TMS-based NIBS plus acupuncture and tDCS plus acupuncture ranked highest for FMA-LE, with ranking probabilities of 89.9 and 85.1%, respectively, followed by tDCS alone (46.5%), acupuncture alone (43.9%), TMS-based NIBS alone (26.9%), and CRT (7.6%). These findings suggest that TMS-based NIBS plus acupuncture and tDCS plus acupuncture may be associated with greater lower limb motor improvement, but the difference between the two combined regimens was not statistically significant and should be interpreted cautiously.

#### 3.5.3 Muscle tone

Eight trials reported MAS scores and involved three interventions (16, 42, 43, 48, 56, 57, 60, 61). The network meta-analysis (Table 4) revealed that TMS-based NIBS combined with acupuncture significantly reduced spasticity compared with TMS-based NIBS alone (MD = −0.61, 95% CI [−0.89 to −0.34]) and acupuncture alone (MD = −0.42, 95% CI [−0.65 to −0.19]). However, the difference between TMS-based NIBS alone and acupuncture alone was not statistically significant (MD = 0.19, 95% CI [−0.15 to 0.53]).

**Table 4:** Network meta-analysis results for MAS [MD (95% CI)].

TMS + ACUP		
−0.61 (−0.89,–0.34)^a^	TMS	
−0.42 (−0.65,–0.19)^a^	0.19 (−0.15,0.53)	ACUP

^a^Indicates a statistically significant comparison, defined as a 95% CI that does not include 0.

According to SUCRA values (Figure 4D), TMS-based NIBS plus acupuncture ranked highest for reducing spasticity, with a ranking probability of 100.0%, followed by acupuncture alone (43.7%) and TMS-based NIBS alone (6.3%). These findings suggest that TMS-based NIBS combined with acupuncture may be associated with greater improvement in muscle tone, although the evidence should be interpreted cautiously because the MAS network included only three intervention nodes.

#### 3.5.4 Balance function

Eight trials reported BBS scores and involved five interventions (22, 23, 26, 30, 40, 45, 65, 66). The network meta-analysis (Table 5) demonstrated that TMS-based NIBS combined with acupuncture significantly improved balance compared with TMS-based NIBS alone (MD = 5.68, 95% CI [4.25 to 7.10]), acupuncture alone (MD = 5.89, 95% CI [3.58 to 8.20]), and CRT (MD = 10.74, 95% CI [8.25 to 13.24]). The difference between TMS-based NIBS plus acupuncture and tDCS plus acupuncture was not statistically significant (MD = 2.97, 95% CI [−0.15 to 6.09]).

**Table 5:** Network meta-analysis results for BBS [MD (95% CI)].

TMS + ACUP				
5.68 (4.25,7.10)^a^	TMS			
5.89 (3.58,8.20)^a^	0.22 (−2.27,2.70)	ACUP		
10.74 (8.25,13.24)^a^	5.07 (2.34,7.80)^a^	4.85 (2.57,7.14)^a^	CRT	
2.97 (−0.15,6.09)	−2.71 (−5.99,0.58)	−2.92 (−5.49,–0.35)^a^	−7.77 (−10.34,–5.21)^a^	tDCS + ACUP

^a^Indicates a statistically significant comparison, defined as a 95% CI that does not include 0.

tDCS combined with acupuncture was significantly more effective than acupuncture alone (MD = −2.92, 95% CI [−5.49 to −0.35]) and CRT (MD = −7.77, 95% CI [−10.34 to −5.21]), but not significantly different from TMS-based NIBS alone (MD = −2.71, 95% CI [−5.99 to 0.58]). According to SUCRA values (Figure 4E), TMS-based NIBS plus acupuncture ranked highest for BBS (99.3%), followed by tDCS plus acupuncture (74.1%), TMS-based NIBS alone (40.5%), acupuncture alone (36.1%), and CRT (0.0%). These findings suggest that TMS-based NIBS plus acupuncture may be associated with greater balance improvement, but the difference between the two combined regimens was not statistically significant and should be interpreted cautiously.

#### 3.5.5 Activities of daily living

Twenty trials reported the BI (23, 27, 31, 32, 36, 40, 41, 57, 59, 63), and Twenty-four trials reported the MBI (16, 25, 26, 33, 34, 37, 39, 40, 42, 43, 45–47, 50–56, 58, 60, 62, 64), covering six interventions in total. The network meta-analysis (Table 6) showed that TMS-based NIBS combined with acupuncture significantly improved ADL compared with TMS-based NIBS alone (MD = 8.33, 95% CI [6.19 to 10.47]), acupuncture alone (MD = 7.95, 95% CI [5.74 to 10.17]), CRT (MD = 12.25, 95% CI [8.73 to 15.78]), and tDCS alone (MD = 12.79, 95% CI [6.34 to 19.24]).

**Table 6:** Network meta-analysis results for ADL [MD (95% CI)].

TMS + ACUP					
8.33 (6.19,10.47) ^a^	TMS				
7.95 (5.74,10.17) ^a^	−0.38 (−3.04,2.29)	ACUP			
12.25 (8.73,15.78)^a^	3.92 (0.03,7.81) ^a^	4.30 (0.57,8.02) ^a^	CRT		
12.79 (6.34,19.24) ^a^	4.46 (−2.16,11.08)	4.84 (−1.33,11.00)	0.54 (−6.28,7.35)	tDCS	
−1.43 (−6.92,4.06)	−9.76 (−15.44,–4.07) ^a^	−9.38 (−14.57,–4.19) ^a^	−13.68 (−19.49,–7.87)^a^	−14.22 (−19.03,–9.41)^a^	tDCS + ACUP

^a^Indicates a statistically significant comparison, defined as a 95% CI that does not include 0.

tDCS combined with acupuncture also showed significantly greater improvement than TMS-based NIBS alone (MD = −9.76, 95% CI [−15.44 to −4.07]), acupuncture alone (MD = −9.38, 95% CI [−14.57 to −4.19]), CRT (MD = −13.68, 95% CI [−19.49 to −7.87]), and tDCS alone (MD = −14.22, 95% CI [−19.03 to −9.41]). However, the difference between TMS-based NIBS plus acupuncture and tDCS plus acupuncture was not statistically significant (MD = −1.43, 95% CI [−6.92 to 4.06]).

According to SUCRA values (Figure 4F), tDCS plus acupuncture ranked highest for ADL (93.8%), followed by TMS-based NIBS plus acupuncture (86.2%), acupuncture alone (50.6%), TMS-based NIBS alone (45.5%), CRT (12.0%), and tDCS alone (12.0%). These findings suggest that both tDCS plus acupuncture and TMS-based NIBS plus acupuncture may be associated with greater improvement in ADL, but their relative difference was not statistically significant. Given that BI and MBI were pooled as ADL outcomes, these results should be interpreted cautiously.

To examine whether pooling BI and MBI influenced the ADL findings, sensitivity analyses were conducted by analyzing BI-only and MBI-only studies separately. In the BI-only analysis, TMS-based NIBS plus acupuncture showed the highest ranking probability, followed by tDCS plus acupuncture and TMS-based NIBS alone. In the MBI-only analysis, tDCS plus acupuncture ranked highest, followed by TMS-based NIBS plus acupuncture. Overall, both scale-specific analyses showed that combined NIBS and acupuncture regimens ranked more favorably than single-modality interventions or CRT, which was generally consistent with the primary pooled ADL analysis. However, the leading combined regimen differed between BI-only and MBI-only analyses, suggesting that the pooled ADL findings should still be interpreted cautiously. The BI-only and MBI-only network plots, SUCRA plots, and comparison-adjusted funnel plots are provided in Supplementary Figures S5–S7.

#### 3.5.6 Safety, dropout, and treatment compliance

Seventeen of the 46 included trials explicitly reported safety or adverse-event information (16, 25, 26, 29–31, 37–39, 43, 44, 49, 50, 55, 56, 60, 63). Most reported no obvious or intervention-related adverse events. When adverse events occurred, they were generally mild and transient, including headache, dizziness, tinnitus, local skin itching, needling-site bleeding, subcutaneous bleeding, local pain, stimulation-site discomfort, nausea, vomiting, rash, and fatigue. One study reported treatment discontinuation due to headache in the tDCS group (55). No consistent evidence suggested an increased risk of serious adverse events with combined NIBS and acupuncture. However, safety outcomes, dropout reasons, and treatment compliance were incompletely reported, and the safety findings should therefore be interpreted cautiously.

#### 3.5.7 Publication bias

**Figure 5:**
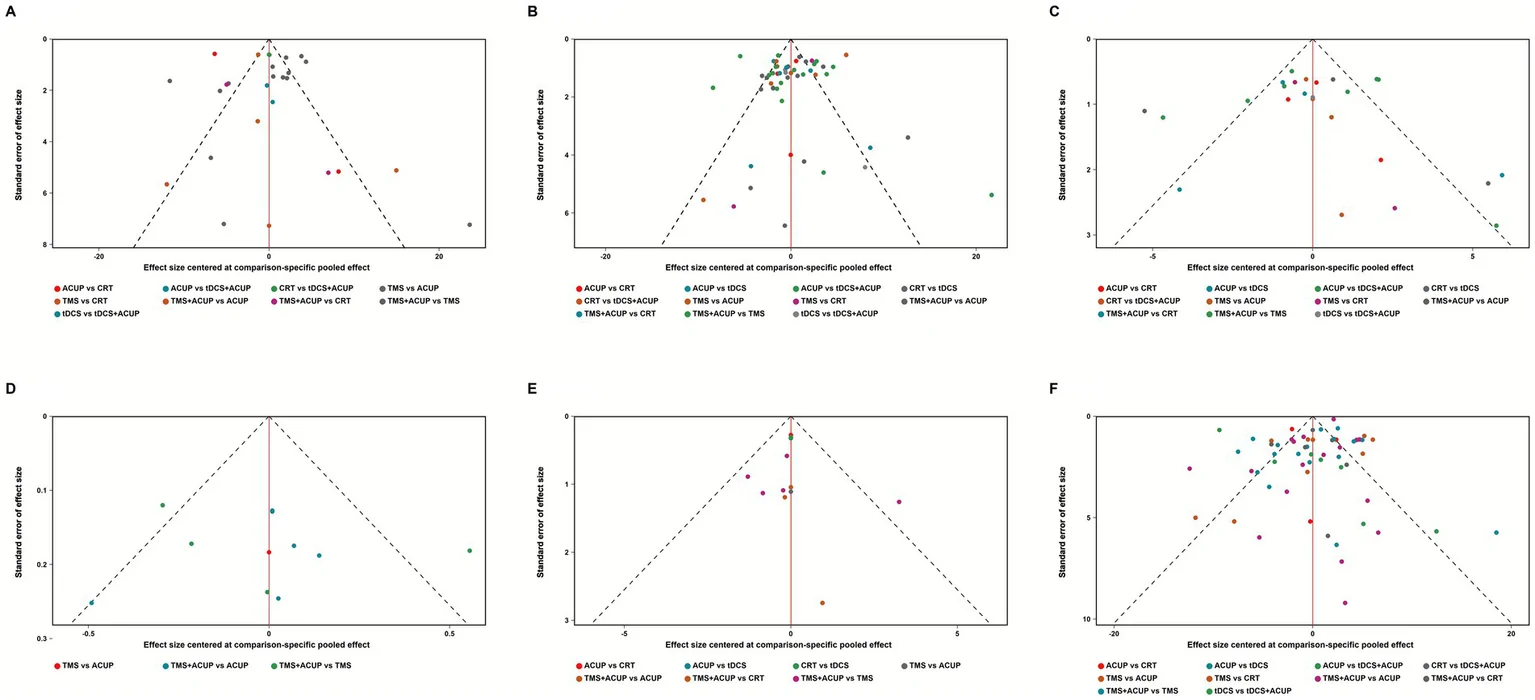
Comparison-adjusted funnel plots for publication bias and small-study effects: **(A)** FMA; **(B)** FMA-UE; **(C)** FMA-LE; **(D)** MAS; **(E)** BBS; **(F)** ADL.

Publication bias and small-study effects were assessed for each outcome via comparison-adjusted funnel plots and Egger’s regression test. Overall, the comparison-adjusted funnel plots were generally centered around the pooled effect estimates, but some dispersion and several outlying studies were observed across outcomes, particularly for FMA, FMA-LE, and ADL (Figure 5). For FMA-UE, most studies were distributed around the center of the funnel plot, although moderate dispersion remained. For MAS and BBS, interpretation of funnel plot asymmetry was limited by the relatively small number of contributing studies.

Egger’s tests were not statistically significant for the assessed outcomes (all *p* > 0.05), providing no strong statistical evidence of small-study effects. However, given the visual dispersion in some funnel plots and the insufficient reporting of allocation concealment and blinding procedures in many included trials, potential publication bias or small-study effects could not be fully excluded. Therefore, the findings should be interpreted with caution.

### 3.6 Confidence in network estimates

The confidence in the network estimates was evaluated using CINeMA, and detailed judgments are presented in Supplementary Table S3. Overall, no comparison was rated as high confidence. Among the 48 evaluated network estimates, 14 were rated as moderate confidence, 23 as low confidence, and 11 as very low confidence. The main reasons for downgrading were within-study bias, imprecision, heterogeneity, and incoherence, whereas indirectness was generally not a major concern. These findings suggest that the estimated effects and treatment rankings should be interpreted cautiously and should not be regarded as definitive evidence of comparative clinical superiority.

## 4 Discussion

### 4.1 Principal findings

This network meta-analysis included 46 RCTs involving 4,080 participants and compared the effects of combined NIBS and acupuncture regimens on post-stroke motor and functional recovery. Overall, combined interventions appeared more beneficial than single-modality therapies or conventional rehabilitation for most outcomes. TMS-based NIBS plus acupuncture and tDCS plus acupuncture showed favorable ranking probabilities across several outcomes. Specifically, tDCS plus acupuncture ranked highly for FMA total, FMA-UE, and ADL, whereas TMS-based NIBS plus acupuncture ranked favorably for FMA-LE, spasticity, and balance. However, differences between these two combined regimens were generally not statistically significant; therefore, the rankings should be interpreted as exploratory probabilities rather than definitive evidence of clinical superiority. The confidence in the network estimates was mostly moderate, low, or very low, and the findings should be interpreted cautiously.

### 4.2 Comparison with previous studies

Previous meta-analyses have suggested that acupuncture combined with rTMS may improve post-stroke upper limb motor dysfunction. Xie et al. reported that acupuncture combined with rTMS improved upper limb motor function, activities of daily living, hemiplegic shoulder pain, and neurophysiological indices, including motor-evoked potential latency, motor-evoked potential amplitude, and central motor conduction time (14). Yan et al. further showed that acupuncture combined with rTMS was superior to acupuncture alone or rTMS alone in improving FMA-UE, MBI, MAS, and NIHSS scores (67). These findings are consistent with our results and support the potential additive value of combining acupuncture with central neuromodulation.

Nevertheless, the present study extends previous evidence in several ways. First, previous reviews mainly focused on rTMS combined with acupuncture and upper limb outcomes (14, 67), whereas our study included multiple NIBS-based combinations, including TMS-based and tDCS based regimens, and evaluated multidimensional outcomes, including global motor function, upper and lower extremity recovery, spasticity, balance, and activities of daily living. Second, previous reviews mainly used pairwise meta-analysis, whereas our network meta-analysis integrated direct and indirect evidence and allowed preliminary comparison and ranking of competing combined regimens. This approach is consistent with recent rehabilitation-related network meta-analyses showing that NMA can help compare multiple treatment options when direct head-to-head trials are limited (68, 69). In addition, evidence from NIBS-related meta-analysis suggests that different stimulation modalities may produce different effects across functional outcomes, highlighting the importance of considering stimulation type, target region, intensity, duration, and timing (70). Therefore, our study adds to the existing literature by framing NIBS combined with acupuncture as a central–peripheral neuromodulatory strategy and by providing preliminary evidence for goal-oriented selection of combined rehabilitation approaches after stroke.

### 4.3 Potential mechanisms across rehabilitation domains

#### 4.3.1 Motor recovery and spasticity: central priming of motor networks

The favorable ranking of TMS-based NIBS plus acupuncture for motor recovery and spasticity may be explained by complementary central and peripheral mechanisms. Post-stroke motor recovery depends on adaptive neuroplasticity, including restoration of cortical excitability, rebalancing of interhemispheric inhibition, strengthening of corticospinal output, and reorganization of motor-related networks (7, 71). NIBS may serve as a form of central priming: high-frequency rTMS or excitatory stimulation can enhance ipsilesional motor cortical excitability, whereas low-frequency rTMS may reduce excessive inhibition from the contralesional hemisphere (72); tDCS may modulate membrane excitability and facilitate activity-dependent plasticity in a polarity- and montage-dependent manner (73). These mechanisms are consistent with current neurorehabilitation theories and rTMS guideline evidence for post-stroke motor recovery.

Acupuncture and electroacupuncture may provide additional peripheral afferent stimulation through somatosensory and proprioceptive pathways (74, 75). Such stimulation may enhance sensorimotor feedback, spinal–cortical communication, and motor relearning, thereby complementing the central priming effects of NIBS. Basic and clinical evidence suggests that acupuncture may influence post-stroke recovery through multiple mechanisms, including modulation of cerebral perfusion, neuroinflammation, neurotrophic signaling, synaptic plasticity, and motor network activity (76). Therefore, the favorable ranking of TMS-based NIBS plus acupuncture for several motor-related outcomes, mainly driven by conventional rTMS studies, may be explained by complementary central and peripheral mechanisms. Specifically, TMS-based NIBS plus acupuncture may improve FMA-related outcomes and spasticity by combining cortical excitability modulation with peripheral afferent input, creating a more favorable neural state for motor relearning and muscle tone regulation.

#### 4.3.2 Balance: multisensory integration and postural control

Balance recovery after stroke involves more than lower limb motor strength. It requires the integration of visual, vestibular, proprioceptive, cerebellar, and cortical motor control systems (77). Stroke survivors frequently present with impaired postural control, asymmetric weight bearing, reduced proprioceptive feedback, and increased dependence on compensatory sensory strategies. Therefore, the favorable ranking of TMS-based NIBS plus acupuncture for BBS may reflect combined effects on motor network excitability and multisensory postural control. Because the TMS-based node was mainly informed by conventional rTMS studies, this interpretation should be understood as being driven primarily by rTMS-based evidence rather than by direct evidence for theta-burst stimulation. rTMS may improve motor network responsiveness and descending control (78), whereas acupuncture may enhance somatosensory input and proprioceptive feedback (79), both of which are important for balance adjustment and postural stability.

This interpretation is consistent with evidence that balance disability is common after stroke and is closely related to impaired postural control and sensory-motor integration (80). Sensory input is increasingly recognized as an active therapeutic target in post-stroke motor rehabilitation, because disrupted sensory processing can limit motor planning, online correction, and functional recovery (81, 82). However, the balance findings in the present study should be interpreted cautiously because the evidence for BBS was relatively limited, and the interpretation of funnel plot asymmetry was constrained by the small number of contributing studies.

#### 4.3.3 ADL: higher-order functional networks and task performance

The finding that tDCS plus acupuncture ranked highest for ADL may reflect the complexity of functional independence after stroke. ADL performance depends not only on limb motor recovery but also on attention, executive function, planning, task sequencing, motivation, balance, and the ability to transfer motor gains into meaningful daily tasks. Compared with rTMS, tDCS may have broader and less focal effects on distributed cortical networks, depending on electrode montage and current flow (83). When combined with acupuncture and rehabilitation training, tDCS may therefore influence both sensorimotor and higher-order functional networks involved in goal-directed behavior and task performance.

This interpretation is supported by evidence that tDCS may improve ADL after stroke, although its effects on limb motor function, muscle strength, and cognitive outcomes remain inconsistent (84). In addition, studies combining tDCS with cognitive or rehabilitation training suggest potential benefits for executive function and instrumental ADL, indicating that tDCS may be particularly relevant when functional independence depends on cognitive-motor integration (85, 86). Nevertheless, in the present network meta-analysis, tDCS plus acupuncture was not statistically superior to TMS-based NIBS plus acupuncture for ADL. Therefore, this result should be interpreted as a potential functional signal rather than definitive evidence that tDCS plus acupuncture is clinically superior to other combined regimen for improving ADL.

From an aging-neuroscience perspective, these findings may be particularly relevant because many stroke survivors are older adults with reduced neuroplastic reserve. Combined NIBS and acupuncture may theoretically target both age- and stroke-related constraints on recovery by modulating cortical excitability while enhancing afferent feedback and sensorimotor integration. However, because most included trials did not stratify outcomes by age or neuroplasticity-related biomarkers, this interpretation remains mechanistic and should be tested in future studies.

### 4.4 Sources of heterogeneity and assumptions of network meta-analysis

The interpretation of the present network estimates should take into account the clinical and methodological diversity of the included trials. Although all studies evaluated combined neuromodulatory rehabilitation after stroke, the enrolled populations were not fully homogeneous. The included trials involved patients with both ischemic and hemorrhagic stroke, covered acute, subacute, and chronic stages, and included participants with different baseline motor impairments. For example, some studies enrolled patients within days or weeks after stroke, whereas others included participants several months after onset. This is clinically important because the response to NIBS and acupuncture may depend on the post-stroke recovery stage and the remaining neuroplastic potential. The intervention protocols were also diverse. Within the TMS-based NIBS node, most evidence was derived from conventional rTMS protocols, particularly 1-Hz low-frequency stimulation over the contralesional M1. However, some trials applied high-frequency stimulation over the ipsilesional M1, bilateral M1, or DLPFC, and a few used iTBS, cTBS, or mixed magnetic stimulation protocols. Because most studies in the TMS-based node used conventional rTMS, the interpretation of this node was mainly driven by rTMS evidence, whereas evidence for iTBS, cTBS, and mixed magnetic stimulation protocols remained sparse. Therefore, separate conclusions regarding theta-burst stimulation cannot be drawn from the current network. Similarly, tDCS studies differed in electrode montage and stimulation targets. The acupuncture component was also not standardized: scalp acupuncture, body acupuncture, electroacupuncture, warm needling, staged acupuncture, movement-related acupuncture, and named traditional protocols such as “Xing Nao Kai Qiao” were all included. These differences are consistent with previous reviews of acupuncture combined with rTMS, which also identified acupoint selection, acupuncture modality, treatment duration, rTMS parameters, and disease stage as major sources of heterogeneity (14).

These variations are directly related to the assumptions of network meta-analysis. The transitivity assumption requires that the main effect modifiers are sufficiently comparable across different treatment comparisons. In this review, the shared stroke rehabilitation context, prespecified treatment nodes, and commonly used outcomes provided a reasonable basis for network synthesis. However, transitivity cannot be fully verified because several important modifiers—including disease stage, baseline severity, stimulation frequency and target, acupuncture modality, treatment duration, and treatment sequence—were not consistently reported or balanced across trials. In addition, some treatment nodes and outcome-specific networks were supported by limited direct evidence. For example, the MAS network involved only three interventions, and the BBS network included relatively few trials. Visual inspection of the comparison-adjusted funnel plots showed some dispersion and outlying studies across several outcomes, particularly for FMA, FMA-LE, and ADL, whereas interpretation for MAS and BBS was limited by the relatively small number of contributing studies. Therefore, although inconsistency was assessed using node-splitting methods when closed loops were available, the absence of statistical inconsistency should not be interpreted as definitive proof of network coherence, especially in sparse networks. Accordingly, SUCRA rankings should be interpreted as exploratory probabilities rather than firm treatment hierarchies. Future head-to-head trials should stratify patients by stroke phase and baseline impairment, standardize NIBS and acupuncture parameters, and report treatment sequence and mechanistic outcomes to strengthen the credibility of indirect comparisons.

### 4.5 Clinical implications for neurorehabilitation and future research

The findings of this study may provide preliminary evidence for goal-oriented selection of combined neuromodulatory strategies in post-stroke rehabilitation. TMS-based NIBS plus acupuncture and tDCS plus acupuncture showed favorable ranking probabilities across several outcomes, but the differences between the two combined regimens were generally not statistically significant. Therefore, these rankings should be interpreted as exploratory probabilities rather than definitive evidence of clinical superiority. TMS-based NIBS plus acupuncture appeared to rank favorably for lower limb motor recovery, spasticity, and balance, whereas tDCS plus acupuncture ranked highly for overall motor function, upper limb motor recovery, and ADL. However, these findings should be viewed as hypothesis-generating rather than direct clinical recommendations. From a mechanistic perspective, combined NIBS and acupuncture may integrate central modulation of cortical excitability with peripheral afferent stimulation and sensorimotor integration, which are both relevant to post-stroke neuroplasticity (87, 88). Nevertheless, current evidence remains insufficient to determine the optimal combined protocol because of variability in stimulation targets, frequency, intensity, acupuncture modality, treatment duration, and treatment sequence. Future large-scale, multicenter, sham-controlled RCTs should standardize stimulation and acupuncture parameters, clarify the timing of NIBS relative to acupuncture and rehabilitation training, and incorporate long-term follow-up, adverse-event reporting, and mechanistic biomarkers such as corticospinal excitability, motor-evoked potentials, functional connectivity, and sensorimotor integration measures.

### 4.6 Strengths and limitations

This study has several strengths. To our knowledge, it is the first systematic review and network meta-analysis to compare multiple combined NIBS and acupuncture regimens for post-stroke rehabilitation. Unlike previous reviews that mainly focused on rTMS combined with acupuncture or on upper limb recovery alone, this review included TMS-based and tDCS-based combinations and evaluated multiple clinically relevant outcomes, including global motor function, upper and lower extremity motor recovery, spasticity, balance, and activities of daily living. The use of network meta-analysis allowed the integration of direct and indirect evidence and provided exploratory rankings of competing combined neuromodulatory strategies. In addition, confidence in the network estimates was assessed using CINeMA, which helped clarify the credibility and limitations of the current findings.

Several limitations should also be acknowledged. First, the risk-of-bias assessment identified several methodological concerns. Although most studies were judged as having a low risk of bias for random sequence generation, incomplete outcome data, selective reporting, and other bias, allocation concealment and blinding procedures were often insufficiently reported. In particular, blinding of participants, personnel, and outcome assessors was frequently unclear, and a small number of studies were judged as high risk in specific domains. These issues may have affected the credibility of the pooled estimates and were therefore considered in the CINeMA assessment. Second, some treatment nodes and outcome-specific comparisons were supported by relatively few direct studies; therefore, SUCRA rankings should be interpreted as probabilities of relative benefit rather than definitive evidence of superiority. Although iTBS, cTBS, and mixed magnetic stimulation protocols were included in the broader TMS-based NIBS node because of sparse evidence, these protocols differ from conventional rTMS in stimulation pattern and physiological dose. Therefore, the findings for the TMS-based node should be interpreted as being driven mainly by conventional rTMS evidence, and separate conclusions regarding theta-burst stimulation cannot be drawn. Third, substantial clinical heterogeneity existed across studies, including differences in NIBS type, stimulation frequency, intensity, target region, treatment duration, treatment sequence, acupuncture modality, acupoint selection, and stimulation dose. These variations limited our ability to determine the optimal combined protocol. Although BI and MBI were pooled because both are commonly used 0–100-point ADL scales, they are not identical instruments. Sensitivity analyses separating BI-only and MBI-only studies suggested broadly consistent directions of effect, but the leading combined regimen differed between scales; therefore, the pooled ADL findings should be interpreted cautiously. Fourth, because many included trials were conducted in clinical settings where acupuncture is routinely integrated into stroke rehabilitation, the applicability of these findings to other health-care systems and rehabilitation contexts requires further validation. Variations in acupuncture traditions, practitioner training, acupoint selection, manipulation techniques, and patient expectations may also have contributed to clinical heterogeneity and may limit generalizability. Fifth, safety outcomes, dropout reasons, and treatment compliance were incompletely reported in many trials, limiting definitive conclusions regarding tolerability. Finally, CINeMA indicated that most network estimates were supported by low or moderate confidence, and several comparisons were rated as very low confidence because of imprecision, heterogeneity, or incoherence. Therefore, the present findings should be interpreted cautiously and considered hypothesis-generating rather than definitive clinical recommendations.

## 5 Conclusion

NIBS combined with acupuncture may provide additional benefits for post-stroke motor and functional recovery compared with single-modality therapy or conventional rehabilitation. TMS-based NIBS plus acupuncture and tDCS plus acupuncture showed favorable ranking probabilities across several outcomes; however, differences between combined regimens were generally not statistically significant. These findings should be interpreted cautiously because of risk-of-bias concerns, substantial clinical heterogeneity, sparse evidence for some comparisons, and limited confidence in several network estimates. Future large-scale, multicenter RCTs with standardized stimulation parameters, acupuncture protocols, sham controls, and mechanistic outcomes are needed.

## Data Availability

The original contributions presented in the study are included in the article/Supplementary material, further inquiries can be directed to the corresponding author/s.

## Glossary

ACUP: acupuncture
ADL: activities of daily living
BBS: Berg Balance Scale
BI: Barthel Index
CINeMA: Confidence in Network Meta-Analysis
CRT: conventional rehabilitation therapy
FMA: Fugl-Meyer Assessment
FMA-LE: Fugl-Meyer Assessment for Lower Extremity
FMA-UE: Fugl-Meyer Assessment for Upper Extremity
MAS: Modified Ashworth Scale
MBI: Modified Barthel Index
MD: mean difference
NIBS: noninvasive brain stimulation
NMA: network meta-analysis
PRISMA: Preferred Reporting Items for Systematic Reviews and Meta-Analyses
RCT: randomized controlled trial
RoB: risk of bias
rTMS: repetitive transcranial magnetic stimulation
SUCRA: surface under the cumulative ranking curve
tDCS: transcranial direct current stimulation
