# Supplementary material for: Combined noninvasive brain stimulation and acupuncture for post-stroke motor recovery: a systematic review and network meta-analysis
Source: Front Neurol. 2026 Aug 20;17:1901205. doi: 10.3389/fneur.2026.1901205 (PMC13538002; doi:10.3389/fneur.2026.1901205)
Supplement: Supplementary file 1 [file Supplementary_file_1.docx]

**Supplementary Table S1.** PRISMA-NMA checklist

| **Section/Topic** | **Item #** | **Checklist Item** | **Reported on Page #** |
| --- | --- | --- | --- |
| **TITLE** |  |  |  |
| Title | 1 | Identify the report as a systematic review *incorporating a network meta-analysis (or related form of meta-analysis).* | Reported in the title: “Combined noninvasive brain stimulation and acupuncture for post-stroke motor recovery: a systematic review and network meta-analysis.” |
|  |  |  |  |
| **ABSTRACT** |  |  |  |
| Structured summary | 2 | Provide a structured summary including, as applicable:  **Background:** main objectives  **Methods:** data sources; study eligibility criteria, participants, and interventions; study appraisal; and *synthesis methods, such as network meta-analysis.*  **Results:** number of studies and participants identified; summary estimates with corresponding confidence/credible intervals; *treatment rankings may also be discussed. Authors may choose to summarize pairwise comparisons against a chosen treatment included in their analyses for brevity.*  **Discussion/Conclusions:** limitations; conclusions and implications of findings.  **Other:** primary source of funding; systematic review registration number with registry name. | Reported in the Abstract: Objective, Methods, Results, and Conclusion. The abstract reports databases searched to July 1, 2026, eligibility, pairwise and frequentist random-effects NMA methods, RoB/CINeMA appraisal, 46 RCTs/4,080 participants, ranking probabilities, main limitations, cautious conclusions, and PROSPERO registration. |
|  |  |  |  |
| **INTRODUCTION** |  |  |  |
| Rationale | 3 | Describe the rationale for the review in the context of what is already known*, including mention of why a network meta-analysis has been conducted.* | Reported in Section 1, Introduction, especially the final two paragraphs explaining the evidence gap and why direct and indirect evidence were integrated using NMA. |
| Objectives | 4 | Provide an explicit statement of questions being addressed, with reference to participants, interventions, comparisons, outcomes, and study design (PICOS). | Reported in Section 1, final paragraph, and Section 2.2, Eligibility criteria, with explicit PICOS elements covering adult post-stroke patients, NIBS plus acupuncture/electroacupuncture, comparators, outcomes, and RCT design. |
|  |  |  |  |
| **METHODS** |  |  |  |
| Protocol and registration | 5 | Indicate whether a review protocol exists and if and where it can be accessed (e.g., Web address); and, if available, provide registration information, including registration number. | Reported in Section 2.1, Protocol registration and reporting standards: PROSPERO registration CRD420251051398, registered on 13 May 2025; PRISMA/PRISMA-NMA reporting standards; checklist identified as Supplementary Table S1. |
| Eligibility criteria | 6 | Specify study characteristics (e.g., PICOS, length of follow-up) and report characteristics (e.g., years considered, language, publication status) used as criteria for eligibility, giving rationale. *Clearly describe eligible treatments included in the treatment network, and note whether any have been clustered or merged into the same node (with justification).* | Reported in Section 2.2, Eligibility criteria, and Section 2.3, Information sources and search strategy. Treatment nodes are defined by active NIBS modality and acupuncture/electroacupuncture combination; low-/high-frequency rTMS, iTBS, cTBS, and mixed magnetic stimulation are justified as a broader TMS-based NIBS node because sparse evidence precluded stable separate nodes. |
| Information sources | 7 | Describe all information sources (e.g., databases with dates of coverage, contact with study authors to identify additional studies) in the search and date last searched. | Reported in Section 2.3: PubMed, Embase, Cochrane Library, Web of Science, Scopus, CNKI, SinoMed, Wanfang, and CQVIP searched from inception to July 1, 2026; manual searches of included-study and review reference lists are also reported. |
| Search | 8 | Present full electronic search strategy for at least one database, including any limits used, such that it could be repeated. | Reported in Section 2.3 and Supplementary Tables S2-S3, which provide full database search strategies, controlled vocabulary terms, free-text terms, search logic, and records retrieved. |
| Study selection | 9 | State the process for selecting studies (i.e., screening, eligibility, included in systematic review, and, if applicable, included in the meta-analysis). | Reported in Section 2.4, Study selection, and Section 3.1/Figure 1. Two reviewers independently screened records, assessed full texts, resolved disagreements, and reported numbers at each stage. |
| Data collection process | 10 | Describe method of data extraction from reports (e.g., piloted forms, independently, in duplicate) and any processes for obtaining and confirming data from investigators. | Reported in Section 2.5, Data collection and management. Two reviewers independently extracted data using a standardized form; missing or ambiguous data were sought from authors or estimated from figures/reported coefficients; data were cross-checked by a third researcher. |
| Data items | 11 | List and define all variables for which data were sought (e.g., PICOS, funding sources) and any assumptions and simplifications made. | Reported in Section 2.5 and Section 2.7. Extracted variables include study characteristics, participants, intervention parameters, outcomes, randomization/allocation/blinding, language, country/region, acupuncture type, NIBS node classification, registration/protocol availability, analysis population, dropout, compliance, adverse events, and outcome-synthesis assumptions such as BI/MBI pooling. |
| **Geometry of the network** | **S1** | Describe methods used to explore the geometry of the treatment network under study and potential biases related to it. This should include how the evidence base has been graphically summarized for presentation, and what characteristics were compiled and used to describe the evidence base to readers. | Reported in Section 2.7, Section 3.5.1, and Figure 3. Network plots are used to summarize direct/indirect comparisons; node size reflects sample size and edge thickness reflects the number of trials. Section 3.5.1 describes dominant nodes, frequent direct comparisons, sparse theta-burst/mixed-protocol evidence, and network structure by outcome. |
| Risk of bias within individual studies | 12 | Describe methods used for assessing risk of bias of individual studies (including specification of whether this was done at the study or outcome level), and how this information is to be used in any data synthesis. | Reported in Section 2.6 and Section 3.3/Figure 2. RoB 1.0 domains, judgment criteria, independent assessment, and use of RoB judgments in interpretation and CINeMA within-study-bias assessment are specified. |
| Summary measures | 13 | State the principal summary measures (e.g., risk ratio, difference in means). *Also describe the use of additional summary measures assessed, such as treatment rankings and surface under the cumulative ranking curve (SUCRA) values, as well as modified approaches used to present summary findings from meta-analyses.* | Reported in Section 2.7. Mean differences with 95% confidence intervals are used; SUCRA values and cumulative ranking plots are reported as relative ranking probabilities, not definitive evidence of superiority. |
| Planned methods of analysis | 14 | Describe the methods of handling data and combining results of studies for each network meta-analysis. This should include, but not be limited to:   - *Handling of multi-arm trials;* - *Selection of variance structure;* - *Selection of prior distributions in Bayesian analyses; and* - *Assessment of model fit.* | Reported in Section 2.7. Pairwise and NMA analyses use Stata 18.0 in a frequentist random-effects framework; league tables, network plots, SUCRA values, comparison-adjusted funnel plots, local inconsistency/incoherence judgments, and transitivity assessment are described. Bayesian priors are not applicable because the analysis is frequentist. |
| **Assessment of Inconsistency** | **S2** | Describe the statistical methods used to evaluate the agreement of direct and indirect evidence in the treatment network(s) studied. Describe efforts taken to address its presence when found. | Reported in Section 2.7. Local inconsistency is considered descriptively based on network structure and CINeMA incoherence judgments; sparse networks and closed-loop limitations are further interpreted in Section 4.4. |
| Risk of bias across studies | 15 | Specify any assessment of risk of bias that may affect the cumulative evidence (e.g., publication bias, selective reporting within studies). | Reported in Section 2.7 and Section 3.5.7/Figure 5. Publication bias and small-study effects are assessed using comparison-adjusted funnel plots and Egger’s regression tests; selective reporting is also considered in the RoB assessment and CINeMA reporting-bias domain. |
| Additional analyses | 16 | Describe methods of additional analyses if done, indicating which were pre-specified. This may include, but not be limited to, the following:   - Sensitivity or subgroup analyses; - Meta-regression analyses; - *Alternative formulations of the treatment network; and* - *Use of alternative prior distributions for Bayesian analyses (if applicable).* | Reported in Section 2.7. Planned additional analyses include subgroup analyses for heterogeneity when sufficient studies are available, BI-only and MBI-only sensitivity analyses for ADL, and descriptive assessment of sparse theta-burst/mixed magnetic stimulation studies. |
|  |  |  |  |
| **RESULTS†** |  |  |  |
| Study selection | 17 | Give numbers of studies screened, assessed for eligibility, and included in the review, with reasons for exclusions at each stage, ideally with a flow diagram. | Reported in Section 3.1 and Figure 1. The manuscript reports 1054 records identified, 461 duplicates removed, 593 records screened, 60 full texts assessed, 14 full-text reports excluded with reasons, and 46 RCTs included; the detailed full-text exclusion list is provided in Supplementary Table S4. |
| **Presentation of network structure** | **S3** | Provide a network graph of the included studies to enable visualization of the geometry of the treatment network. | Reported in Section 3.5.1 and Figure 3. Network graphs are provided for FMA total, FMA-UE, FMA-LE, MAS, BBS, and ADL. |
| **Summary of network geometry** | **S4** | Provide a brief overview of characteristics of the treatment network. This may include commentary on the abundance of trials and randomized patients for the different interventions and pairwise comparisons in the network, gaps of evidence in the treatment network, and potential biases reflected by the network structure. | Reported in Section 3.5.1. The manuscript summarizes node contributions, most frequent direct comparisons, dominance of TMS-based NIBS plus acupuncture/TMS-based NIBS/acupuncture-alone nodes, sparse theta-burst or mixed magnetic stimulation evidence, and outcome-specific network structures. |
| Study characteristics | 18 | For each study, present characteristics for which data were extracted (e.g., study size, PICOS, follow-up period) and provide the citations. | Reported in Section 3.2 and Supplementary Table S5. Study size, patient characteristics, stroke type/stage, intervention characteristics, comparators, outcomes, country/region/language concentration, and citations are summarized. |
| Risk of bias within studies | 19 | Present data on risk of bias of each study and, if available, any outcome level assessment. | Reported in Section 3.3 and Figure 2. The manuscript presents domain-level RoB results, including random sequence generation, allocation concealment, performance bias, detection bias, attrition bias, selective reporting, and other bias. |
| Results of individual studies | 20 | For all outcomes considered (benefits or harms), present, for each study: 1) simple summary data for each intervention group, and 2) effect estimates and confidence intervals. *Modified approaches may be needed to deal with information from larger networks.* | Reported across Supplementary Table S5, Supplementary Figures 1-4, and main-text Tables 1-6. Study characteristics and available study-level data are provided in the supplementary material, while network effect estimates with 95% CIs are summarized by outcome in Tables 1-6. |
| Synthesis of results | 21 | Present results of each meta-analysis done, including confidence/credible intervals. *In larger networks, authors may focus on comparisons versus a particular comparator (e.g. placebo or standard care), with full findings presented in an appendix. League tables and forest plots may be considered to summarize pairwise comparisons.* If additional summary measures were explored (such as treatment rankings), these should also be presented. | Reported in Sections 3.4-3.5. Pairwise results are summarized in Section 3.4 and Supplementary Figures 1-4; NMA results are reported for FMA total, FMA-UE, FMA-LE, MAS, BBS, and ADL in Tables 1-6 with MDs and 95% CIs; SUCRA rankings are shown in Figure 4. |
| **Exploration for inconsistency** | **S5** | Describe results from investigations of inconsistency. This may include such information as measures of model fit to compare consistency and inconsistency models, *P* values from statistical tests, or summary of inconsistency estimates from different parts of the treatment network. | Reported in Section 3.6 and Section 4.4. CINeMA incoherence judgments are summarized with confidence ratings, and the Discussion interprets consistency/inconsistency cautiously, noting that sparse networks and limited closed loops mean absence of statistical inconsistency should not be treated as definitive proof of coherence. |
| Risk of bias across studies | 22 | Present results of any assessment of risk of bias across studies for the evidence base being studied. | Reported in Section 3.5.7 and Figure 5. Comparison-adjusted funnel plots and Egger’s tests are presented; the manuscript concludes that potential publication bias or small-study effects cannot be fully excluded. |
| Results of additional analyses | 23 | Give results of additional analyses, if done (e.g., sensitivity or subgroup analyses, meta-regression analyses*, alternative network geometries studied, alternative choice of prior distributions for Bayesian analyses,* and so forth). | Reported in Section 3.4 and Section 3.5.5. Subgroup analyses for pairwise heterogeneity are summarized in Section 3.4; BI-only and MBI-only ADL sensitivity analyses are reported in Section 3.5.5 and Supplementary Figures 5-7. |
|  |  |  |  |
| **DISCUSSION** |  |  |  |
| Summary of evidence | 24 | Summarize the main findings, including the strength of evidence for each main outcome; consider their relevance to key groups (e.g., healthcare providers, users, and policy-makers). | Reported in Section 4, Principal findings, and Section 3.6. Main findings are summarized with attention to outcome-specific rankings, non-significant differences between combined regimens, and CINeMA confidence ratings showing no high-confidence evidence. |
| Limitations | 25 | Discuss limitations at study and outcome level (e.g., risk of bias), and at review level (e.g., incomplete retrieval of identified research, reporting bias). *Comment on the validity of the assumptions, such as transitivity and consistency. Comment on any concerns regarding network geometry (e.g., avoidance of certain comparisons).* | Reported in Section 4.4 and Section 4.6. Limitations address study-level RoB, sparse nodes, heterogeneity, BI/MBI pooling, regional/language concentration of acupuncture evidence, incomplete safety/dropout/compliance reporting, confidence limitations, and assumptions of transitivity/consistency/network geometry. |
| Conclusions | 26 | Provide a general interpretation of the results in the context of other evidence, and implications for future research. | Reported in Conclusions and Section 4.5. The manuscript provides cautious interpretation, avoids direct clinical recommendations, and identifies future research needs including multicenter sham-controlled RCTs, standardized protocols, long-term follow-up, adverse-event reporting, and mechanistic biomarkers. |
|  |  |  |  |
| **FUNDING** |  |  |  |
| Funding | 27 | Describe sources of funding for the systematic review and other support (e.g., supply of data); role of funders for the systematic review. This should also include information regarding whether funding has been received from manufacturers of treatments in the network and/or whether some of the authors are content experts with professional conflicts of interest that could affect use of treatments in the network. | Reported in the Funding and Conflict of interest sections. The manuscript identifies National Natural Science Foundation of China Regional Joint Fund support (Grant No. U23A20506), states the funder had no role in the study, and declares no commercial or financial conflicts of interest. |

PICOS = population, intervention, comparators, outcomes, study design.

* Text in italics indicateS wording specific to reporting of network meta-analyses that has been added to guidance from the PRISMA statement.

† Authors may wish to plan for use of appendices to present all relevant information in full detail for items in this section.

Supplementary Table S2. Search strategies for English Database

| **1A: Search Strategy for PubMed** | | | |
| --- | --- | --- | --- |
| **No.** | **Search Terms** | **Search Logic** | **Results** |
| #1 | "Stroke"[MeSH Terms] OR "Brain Ischemia"[MeSH Terms] OR "Cerebral Infarction"[MeSH Terms] OR "Intracranial Hemorrhages"[MeSH Terms] OR stroke[Title/Abstract] OR poststroke[Title/Abstract] OR post-stroke[Title/Abstract] OR "cerebrovascular accident"[Title/Abstract] OR "cerebral infarction"[Title/Abstract] OR "brain infarction"[Title/Abstract] OR "cerebral hemorrhage"[Title/Abstract] OR "intracerebral hemorrhage"[Title/Abstract] | Stroke-related MeSH and free-text terms | 550314 |
| #2 | "Transcranial Magnetic Stimulation"[MeSH Terms] OR "Transcranial Direct Current Stimulation"[MeSH Terms] OR "repetitive transcranial magnetic stimulation"[Title/Abstract] OR rTMS[Title/Abstract] OR "transcranial direct current stimulation"[Title/Abstract] OR tDCS[Title/Abstract] OR "theta burst stimulation"[Title/Abstract] OR "intermittent theta burst stimulation"[Title/Abstract] OR iTBS[Title/Abstract] | Noninvasive brain stimulation-related MeSH and free-text terms | 32602 |
| #3 | "Acupuncture Therapy"[MeSH Terms] OR "Acupuncture"[MeSH Terms] OR "Electroacupuncture"[MeSH Terms] OR acupuncture[Title/Abstract] OR electroacupuncture[Title/Abstract] OR electro-acupuncture[Title/Abstract] OR "scalp acupuncture"[Title/Abstract] OR "body acupuncture"[Title/Abstract] | Acupuncture-related MeSH and free-text terms | 45860 |
| #4 | "motor function"[Title/Abstract] OR "motor recovery"[Title/Abstract] OR "upper limb"[Title/Abstract] OR "lower limb"[Title/Abstract] OR spasticity[Title/Abstract] OR balance[Title/Abstract] OR gait[Title/Abstract] OR "activities of daily living"[Title/Abstract] OR ADL[Title/Abstract] OR FMA[Title/Abstract] OR "Fugl-Meyer"[Title/Abstract] OR MAS[Title/Abstract] OR "Modified Ashworth"[Title/Abstract] OR BBS[Title/Abstract] OR "Berg Balance Scale"[Title/Abstract] OR BI[Title/Abstract] OR "Barthel Index"[Title/Abstract] OR MBI[Title/Abstract] OR "Modified Barthel Index"[Title/Abstract] | Motor and functional outcome-related terms | 712777 |
| #5 | "Randomized Controlled Trial"[Publication Type] OR "Random Allocation"[MeSH Terms] OR "Controlled Clinical Trial"[Publication Type] OR random*[Title/Abstract] OR randomized[Title/Abstract] OR randomised[Title/Abstract] OR randomly[Title/Abstract] OR RCT[Title/Abstract] | Randomized or controlled trial-related terms | 2064223 |
| #6 | #1 AND #2 AND #3 AND #4 AND #5 | Final combined search query | 34 |
| **1B: Search Strategy for EMBASE** | | | |
| **No.** | **Search Terms** | **Search Logic** | **Results** |
| #1 | 'stroke'/exp OR 'brain ischemia'/exp OR 'cerebral infarction'/exp OR 'intracranial hemorrhage'/exp OR stroke:ti,ab,kw OR poststroke:ti,ab,kw OR 'post-stroke':ti,ab,kw OR 'cerebrovascular accident':ti,ab,kw OR 'cerebral infarction':ti,ab,kw OR 'brain infarction':ti,ab,kw OR 'cerebral hemorrhage':ti,ab,kw OR 'intracerebral hemorrhage':ti,ab,kw | Stroke-related MeSH and free-text terms | 1058850 |
| #2 | 'transcranial magnetic stimulation'/exp OR 'transcranial direct current stimulation'/exp OR 'repetitive transcranial magnetic stimulation':ti,ab,kw OR rtms:ti,ab,kw OR 'transcranial direct current stimulation':ti,ab,kw OR tdcs:ti,ab,kw OR 'theta burst stimulation':ti,ab,kw OR 'intermittent theta burst stimulation':ti,ab,kw OR itbs:ti,ab,kw | Noninvasive brain stimulation-related MeSH and free-text terms | 63128 |
| #3 | 'acupuncture'/exp OR 'electroacupuncture'/exp OR acupuncture:ti,ab,kw OR electroacupuncture:ti,ab,kw OR 'electro-acupuncture':ti,ab,kw OR 'scalp acupuncture':ti,ab,kw OR 'body acupuncture':ti,ab,kw | Acupuncture-related MeSH and free-text terms | 75440 |
| #4 | 'motor function':ti,ab,kw OR 'motor recovery':ti,ab,kw OR 'upper limb':ti,ab,kw OR 'lower limb':ti,ab,kw OR spasticity:ti,ab,kw OR balance:ti,ab,kw OR gait:ti,ab,kw OR 'activities of daily living':ti,ab,kw OR adl:ti,ab,kw OR fma:ti,ab,kw OR 'fugl-meyer':ti,ab,kw OR mas:ti,ab,kw OR 'modified ashworth':ti,ab,kw OR bbs:ti,ab,kw OR 'berg balance scale':ti,ab,kw OR bi:ti,ab,kw OR 'barthel index':ti,ab,kw OR mbi:ti,ab,kw OR 'modified barthel index':ti,ab,kw | Motor and functional outcome-related terms | 988181 |
| #5 | 'randomized controlled trial'/exp OR 'randomization'/exp OR 'controlled clinical trial'/exp OR random*:ti,ab,kw OR randomized:ti,ab,kw OR randomised:ti,ab,kw OR randomly:ti,ab,kw OR rct:ti,ab,kw | Randomized or controlled trial-related terms | 3068197 |
| #6 | #1 AND #2 AND #3 AND #4 AND #5 | Final combined search query | 70 |
| **1C: Search Strategy for web of science** | | | |
| **No.** | **Search Terms** | **Search Logic** | **Results** |
| #1 | TS=("stroke" OR "poststroke" OR "post-stroke" OR "cerebrovascular accident" OR "cerebral infarction" OR "brain infarction" OR "cerebral hemorrhage" OR "intracerebral hemorrhage") | Stroke-related terms in the topic field | 517549 |
| #2 | TS=("repetitive transcranial magnetic stimulation" OR rTMS OR "transcranial direct current stimulation" OR tDCS OR "theta burst stimulation" OR "intermittent theta burst stimulation" OR iTBS) | Noninvasive brain stimulation-related terms in the topic field | 27601 |
| #3 | TS=(acupuncture OR electroacupuncture OR "electro-acupuncture" OR "scalp acupuncture" OR "body acupuncture") | Acupuncture-related terms in the topic field | 31686 |
| #4 | TS=("motor function" OR "motor recovery" OR "upper limb" OR "lower limb" OR spasticity OR balance OR gait OR "activities of daily living" OR ADL OR FMA OR "Fugl-Meyer" OR MAS OR "Modified Ashworth" OR BBS OR "Berg Balance Scale" OR BI OR "Barthel Index" OR MBI OR "Modified Barthel Index") | Motor and functional outcome-related terms in the topic field | 1723342 |
| #5 | TS=(random* OR randomized OR randomised OR randomly OR RCT) | Randomized or controlled trial-related terms in the topic field | 2666855 |
| #6 | #1 AND #2 AND #3 AND #4 AND #5 | Final combined search query | 38 |
| Index: SCI-EXPANDED, SSCI, CPCI-S, ESCI | | | |
| **1D: Search Strategy for Cochrane Library** | | | |
| **No.** | **Search Terms** | **Search Logic** | **Results** |
| #1 | [mh "Stroke"] OR [mh "Brain Ischemia"] OR [mh "Cerebral Infarction"] OR [mh "Intracranial Hemorrhages"] | MeSH terms for stroke | 23689 |
| #2 | stroke:ti,ab,kw OR poststroke:ti,ab,kw OR "post-stroke":ti,ab,kw OR "cerebrovascular accident":ti,ab,kw OR "cerebral infarction":ti,ab,kw OR "brain infarction":ti,ab,kw OR "cerebral hemorrhage":ti,ab,kw OR "intracerebral hemorrhage":ti,ab,kw | Free-text stroke-related terms | 91088 |
| #3 | #1 OR #2 | Combined stroke-related terms | 93088 |
| #4 | [mh "Transcranial Magnetic Stimulation"] OR [mh "Transcranial Direct Current Stimulation"] | MeSH terms for noninvasive brain stimulation | 5831 |
| #5 | "repetitive transcranial magnetic stimulation":ti,ab,kw OR rTMS:ti,ab,kw OR "transcranial direct current stimulation":ti,ab,kw OR tDCS:ti,ab,kw OR "theta burst stimulation":ti,ab,kw OR "intermittent theta burst stimulation":ti,ab,kw OR iTBS:ti,ab,kw | Free-text noninvasive brain stimulation-related terms | 16720 |
| #6 | #4 OR #5 | Combined noninvasive brain stimulation-related terms | 17548 |
| #7 | [mh "Acupuncture Therapy"] OR [mh "Acupuncture"] OR [mh "Electroacupuncture"] | MeSH terms for acupuncture | 7798 |
| #8 | acupuncture:ti,ab,kw OR electroacupuncture:ti,ab,kw OR "electro-acupuncture":ti,ab,kw OR "scalp acupuncture":ti,ab,kw OR "body acupuncture":ti,ab,kw | Free-text acupuncture-related terms | 24979 |
| #9 | #7 OR #8 | Combined acupuncture-related terms | 25191 |
| #10 | "motor function":ti,ab,kw OR "motor recovery":ti,ab,kw OR "upper limb":ti,ab,kw OR "lower limb":ti,ab,kw OR spasticity:ti,ab,kw OR balance:ti,ab,kw OR gait:ti,ab,kw OR "activities of daily living":ti,ab,kw OR ADL:ti,ab,kw OR FMA:ti,ab,kw OR "Fugl-Meyer":ti,ab,kw OR MAS:ti,ab,kw OR "Modified Ashworth":ti,ab,kw OR BBS:ti,ab,kw OR "Berg Balance Scale":ti,ab,kw OR BI:ti,ab,kw OR "Barthel Index":ti,ab,kw OR MBI:ti,ab,kw OR "Modified Barthel Index":ti,ab,kw | Motor and functional outcome-related terms | 116427 |
| #11 | random*:ti,ab,kw OR randomized:ti,ab,kw OR randomised:ti,ab,kw OR randomly:ti,ab,kw OR RCT:ti,ab,kw | Randomized or controlled trial-related terms | 1518934 |
| #12 | #3 AND #6 AND #9 AND #10 AND #11 | Final combined search query | 38 |
| **1E: Search Strategy for Scopus** | | | |
| **No.** | **Search Terms** | **Search Logic** | **Results** |
| #1 | TITLE-ABS-KEY ("stroke" OR "poststroke" OR "post-stroke" OR "cerebrovascular accident" OR "cerebral infarction" OR "brain infarction" OR "cerebral hemorrhage" OR "intracerebral hemorrhage") | Stroke-related terms in title, abstract, and keywords | 804913 |
| #2 | TITLE-ABS-KEY ("repetitive transcranial magnetic stimulation" OR rTMS OR "transcranial direct current stimulation" OR tDCS OR "theta burst stimulation" OR "intermittent theta burst stimulation" OR iTBS) | Noninvasive brain stimulation-related terms in title, abstract, and keywords | 31373 |
| #3 | TITLE-ABS-KEY (acupuncture OR electroacupuncture OR "electro-acupuncture" OR "scalp acupuncture" OR "body acupuncture") | Acupuncture-related terms in title, abstract, and keywords | 71216 |
| #4 | TITLE-ABS-KEY ("motor function" OR "motor recovery" OR "upper limb" OR "lower limb" OR spasticity OR balance OR gait OR "activities of daily living" OR ADL OR FMA OR "Fugl-Meyer" OR MAS OR "Modified Ashworth" OR BBS OR "Berg Balance Scale" OR BI OR "Barthel Index" OR MBI OR "Modified Barthel Index") | Motor and functional outcome-related terms in title, abstract, and keywords | 2273301 |
| #5 | TITLE-ABS-KEY (random* OR randomized OR randomised OR randomly OR RCT) | Randomized or controlled trial-related terms in title, abstract, and keywords | 3922746 |
| #6 | #1 AND #2 AND #3 AND #4 AND #5 | Final combined search query | 60 |

### Note: Search time range for all tables is from inception to 01 July 2026.

Supplementary Table S3. Search strategies for Chinese Database

| **1A:China National Knowledge Infrastructure** | | |
| --- | --- | --- |
| **No.** | **Search Terms** | **Results** |
| 1（P） | (TKA=中风 OR TKA=卒中 OR TKA=脑血管 OR TKA=脑梗塞 OR TKA=脑梗死 OR TKA=脑血栓 OR TKA=脑栓塞 OR TKA=脑出血 OR TKA=脑溢血 OR TKA=蛛网膜下腔出血) | 639,869 |
| 2（I） | (TKA=脑刺激 OR TKA=经颅直流电 OR TKA=直流电刺激 OR TKA=经颅磁刺激 OR TKA=重复经颅磁 OR TKA=非侵入性脑刺激技术 OR TKA=无创脑刺激技术 OR TKA=无创神经调控技术 OR TKA=TMS OR TKA=tDCS OR TKA=TBS) | 49,322 |
| 3（I） | (TKA=针灸 OR TKA=针刺 OR TKA=电针 OR TKA=火针 OR TKA=头针 OR TKA=手捻针 OR TKA=针刀 OR TKA=头皮针 OR TKA=针法 OR TKA=毫针 OR TKA=体针 OR TKA=温针) | 300,111 |
| 4（O） | (TKA=运动功能 OR TKA=上肢 OR TKA=下肢 OR TKA=步态 OR TKA=平衡) | 559,214 |
| 5（C/S） | (TKA=随机 OR TKA=盲法 OR TKA=单盲 OR TKA=双盲 OR TKA=三盲 OR TKA=安慰剂) | 2,241,992 |
| 6（P&I） |  | 550 |
| 7（P&I&O） |  | 191 |
| 8 （PICOS） |  | 94 |
| **1B:Chinese Biological Medicine Database** | | |
| **No.** | **Search Terms** | **Results** |
| 1（P） | 中风 OR 卒中 OR 脑血管 OR 脑梗塞 OR 脑梗死 OR 脑血栓 OR 脑栓塞 OR 脑出血 OR 脑溢血 OR 蛛网膜下腔出血 OR "卒中"[不加权:扩展] OR "颅内栓塞和血栓形成"[不加权:扩展] OR "脑出血"[不加权:扩展] OR "蛛网膜下腔出血"[不加权:扩展] | 485881 |
| 2（I） | 脑刺激 OR 经颅直流电 OR 直流电刺激 OR 经颅磁刺激 OR 重复经颅磁 OR 非侵入性脑刺激 OR 无创脑刺激 OR 无创神经调控 OR TMS OR "经颅直流电刺激"[不加权:扩展] OR "经颅磁刺激"[不加权:扩展] | 10517 |
| 3（I） | 针灸 OR 针刺 OR 电针 OR 火针 OR 头针 OR 针刀 OR 头皮针 OR 针法 OR 毫针 OR 体针 OR 温针 OR "针灸疗法"[不加权:扩展] OR "针刀疗法"[不加权:扩展] OR "毫针"[不加权:扩展] | 340783 |
| 4（O） | 运动功能 OR 上肢 OR 下肢 OR 步态 OR 平衡 OR "运动障碍"[不加权:扩展] OR "上肢"[不加权:扩展] OR "下肢"[不加权:扩展] OR "步态"[不加权:扩展] OR "姿势平衡"[不加权:扩展] | [47](javascript:void(0);)1610 |
| 5（C/S） | 随机 OR 盲法 OR 单盲 OR 双盲 OR 三盲 OR 安慰剂 OR "随机对照试验"[不加权:扩展] OR "单盲法"[不加权:扩展] OR "双盲法"[不加权:扩展] OR "安慰剂"[不加权:扩展] | 2194543 |
| 6（P&I） |  | 975 |
| 7（P&I&O） |  | 296 |
| 8 （PICOS） |  | 216 |
| **1C:Chinese Science and Technique Journals Database（VIP）** | | |
| **No.** | **Search Terms** | **Results** |
| 1（P） | U=卒中 OR U=中风 OR U=脑血管 OR U=脑梗塞 OR U=脑血栓 OR U=脑栓塞 OR U=脑出血 OR U=脑溢血 OR U=蛛网膜下腔出血 | 548,794 |
| 2（I） | U=针灸 OR U=针刺 OR U=电针 OR U=火针 OR U=头针 OR U=针刀 OR U=手捻针 OR U=头皮针 OR U=针法 OR U=毫针 OR U=体针 OR U=温针 | 380,170 |
| 3（I） | U=tDCS OR U=TMS OR U=脑刺激 OR U=经颅直流电 OR U=直流电刺激 OR U=经颅磁刺激 OR U=重复经颅磁 OR U=非侵入性脑刺激技术 OR U=无创神经调控技术 OR U=无创脑刺激技术 | 88,160 |
| 4（O） | U=运动功能 OR U=上肢 OR U=下肢 OR U=步态 OR U=平衡 | 1,183,334 |
| 5（C/S） | U=随机 OR U=盲法 OR U=单盲 OR U=双盲 OR U=三盲 OR U=安慰剂 | 2,770,450 |
| 6（P&I） |  | 1,380 |
| 7（P&I&O） |  | 476 |
| 8 （PICOS） |  | 348 |
| **1D:Wanfang Database** | | |
| **No.** | **Search Terms** | **Results** |
| 1（P） | "卒中" OR "中风" OR "脑血管" OR "脑梗塞" OR "脑血栓" OR "脑栓塞" OR "脑出血" OR "脑溢血" OR "蛛网膜下腔出血" | 1,450,665 |
| 2（I） | "针灸" OR "针刺" OR "电针" OR "火针" OR "头针" OR "针刀" OR "手捻针" OR "头皮针" OR "针法" OR "毫针" OR "体针" OR "温针" | 488,542 |
| 3（I） | "tDCS" OR "TMS" OR "脑刺激" OR "经颅直流电" OR "直流电刺激" OR "经颅磁刺激" OR "重复经颅磁" OR "非侵入性脑刺激技术" OR "无创神经调控技术" OR "无创脑刺激技术" | 152,303 |
| 4（O） | "运动功能" OR "上肢" OR "下肢" OR "步态" OR "平衡" | 3,376,663 |
| 5（C/S） | "随机" OR "盲法" OR "单盲" OR "双盲" OR "三盲"OR "安慰剂" | 3,931,950 |
| 6（P&I） |  | 568 |
| 7（P&I&O） |  | 189 |
| 8 （PICOS） |  | 156 |

**Supplementary Table S4.** Full-text reports excluded with reasons

| **No.** | **Study** | **Title** | **Reason for exclusion** | **Specific reason** |
| --- | --- | --- | --- | --- |
| 1 | **Zheng et al., 2013** | Effects of stage-specific acupuncture combined with repetitive transcranial magnetic stimulation on quality of life in patients with stroke | Duplicate publication | This report was a duplicate publication of “Effects of repetitive transcranial magnetic stimulation combined with acupuncture on plasma NSE and MRS in patients with cerebral infarction.” |
| 2 | **Cheng et al., 2015** | Clinical comparative study of transcranial direct current stimulation and electroacupuncture for upper-limb spasticity after stroke | Ineligible intervention | The study compared transcranial direct current stimulation with electroacupuncture and did not include an eligible group receiving the combined intervention. |
| 3 | **Qin et al., 2018** | Effects of repetitive transcranial magnetic stimulation at scalp acupoints on spastic upper-limb hemiplegia after stroke | Ineligible intervention | The stimulation target was a scalp acupoint rather than a prespecified conventional cortical target. |
| 4 | **Hathaiareerug et al., 2019** | Comparison between transcranial direct current stimulation and acupuncture on upper extremity rehabilitation in stroke: a single-blind randomized controlled trial | Intervention mismatch | The study compared tDCS plus conventional rehabilitation with electro-acupuncture plus conventional rehabilitation. It did not evaluate a combined NIBS plus acupuncture/electroacupuncture intervention against an eligible comparator, and therefore could not be assigned to the prespecified combined-treatment network nodes. |
| 5 | **Cao et al., 2022** | Kaiqiaotongluo acupuncture combined with repetitive transcranial magnetic stimulation for the treatment of hand dysfunction after stroke | Outcome not eligible for the main quantitative synthesis | The study focused on post-stroke hand dysfunction and assessed the Broetz hand function scale, hemiplegic hand function grading, and MBI. It did not report the prespecified main motor, spasticity, or balance outcomes required for network classification, such as FMA, MAS, or BBS. Although MBI was reported, the hand-specific primary outcomes were not eligible for the main network synthesis. |
| 6 | **Lei et al., 2024** | Effects of acupuncture combined with repetitive transcranial magnetic stimulation and deep muscle stimulation on upper-limb spasticity and cortical oxygenation after stroke | Ineligible intervention | The combined group additionally received deep muscle stimulation, so the effect of acupuncture combined with repetitive transcranial magnetic stimulation could not be isolated. |
| 7 | **Wu et al., 2024** | Effect of MEP-oriented scalp acupuncture combined with transcranial magnetic stimulation on limb motor ability in patients with ischemic stroke hemiplegia | Ineligible intervention | Both groups received combined scalp acupuncture and transcranial magnetic stimulation. The comparison evaluated different acupoint-selection methods rather than the effect of the eligible combined intervention. |
| 8 | **Xu et al., 2024** | Effects of Acupuncture Combined with Transcranial Direct Current Stimulation on Hand Dysfunction in Patients with Acute Cerebral Infarction | Ineligible outcome | None of the outcomes prespecified for this review were reported. |
| 9 | **Chen et al., 2025** | Effect of point-toward-point acupuncture with elongated needles on lower-limb function in patients with post-stroke foot drop | Ineligible intervention | The study compared elongated-needle acupuncture, electromyographic biofeedback, and transcranial magnetic stimulation as separate interventions and did not include an eligible combined-intervention group. |
| 10 | **Guo et al., 2025** | Effects of transcranial alternating current stimulation combined with electroacupuncture in patients with post-stroke attention impairment | Ineligible outcome | None of the outcomes prespecified for this review were reported. |
| 11 | **Wan et al., 2025** | Effects of warm-needle acupuncture combined with repetitive transcranial magnetic stimulation on limb dysfunction after stroke | Ineligible study design | Group allocation was not random. |
| 12 | **Zhao et al., 2025** | Clinical efficacy of acupuncture combined with transcranial direct current stimulation for post-stroke depression and its effects on serum RBP4 and Lp-PLA2 levels | Unclear outcome reporting | The Fugl-Meyer Assessment outcome was described incorrectly or inconsistently, preventing reliable interpretation and data extraction. |
| 13 | **Zhou et al., 2025** | A clinical study on auricular acupressure combined with repetitive transcranial magnetic stimulation (rTMS) in the treatment of lower limb spastic hemiplegia after ischemic stroke | Conference abstract only; insufficient data for quantitative synthesis | Only a conference abstract was available. Although the report described randomization and relevant outcomes, including MAS, CSI, FMA-LE, FAC, BBS, and MBI, group-specific numerical data required for meta-analysis, such as means with standard deviations or change scores, were not available, and the full-text article could not be obtained. |
| 14 | **Li et al., 2026** | Clinical observation of warm-needle acupuncture combined with transcranial magnetic stimulation for postoperative hemiplegia after cerebral hemorrhage | Unclear outcome reporting | The Fugl-Meyer Assessment outcome was described incorrectly or inconsistently, preventing reliable interpretation and data extraction. |

*Abbreviations: BBS, Berg Balance Scale; CSI, Composite Spasticity Index; DMS, deep muscle stimulation; FAC, Functional Ambulation Category; FMA, Fugl-Meyer Assessment; FMA-LE, Fugl-Meyer Assessment of the lower extremity; MAS, Modified Ashworth Scale; MBI, Modified Barthel Index; MEP, motor evoked potential; MRS, magnetic resonance spectroscopy; NIBS, non-invasive brain stimulation; NSE, neuron-specific enolase; rTMS, repetitive transcranial magnetic stimulation; tDCS, transcranial direct current stimulation.*

**Supplementary Table S5.** Confidence in network estimates assessed using CINeMA

| **Outcome** | **Comparison** | **Within-study bias** | **Reporting bias** | **Indirectness** | **Imprecision** | **Heterogeneity** | **Incoherence** | **Overall confidence** |
| --- | --- | --- | --- | --- | --- | --- | --- | --- |
| FMA | ACUP:TMS + ACUP | Some concerns | Low risk | No concerns | Major concerns | Major concerns | No concerns | Very low |
|  | TMS:TMS + ACUP | Some concerns | Low risk | No concerns | No concerns | Major concerns | No concerns | Low |
|  | ACUP:tDCS + ACUP | Some concerns | Low risk | No concerns | Major concerns | No concerns | Major concerns | Very low |
|  | CRT:tDCS + ACUP | Some concerns | Low risk | No concerns | No concerns | No concerns | Major concerns | Low |
|  | tDCS:tDCS + ACUP | Some concerns | Low risk | No concerns | No concerns | No concerns | Major concerns | Low |
|  | ACUP:CRT | Some concerns | Low risk | No concerns | No concerns | No concerns | Major concerns | Low |
|  | ACUP:TMS | Some concerns | Low risk | No concerns | Major concerns | No concerns | No concerns | Low |
| FMA-UE | ACUP:TMS + ACUP | Some concerns | Low risk | No concerns | No concerns | No concerns | No concerns | Moderate |
|  | TMS:TMS + ACUP | Some concerns | Low risk | No concerns | No concerns | No concerns | No concerns | Moderate |
|  | CRT:TMS + ACUP | Some concerns | Low risk | No concerns | No concerns | No concerns | No concerns | Moderate |
|  | ACUP:tDCS + ACUP | Some concerns | Low risk | No concerns | No concerns | No concerns | No concerns | Moderate |
|  | CRT:tDCS + ACUP | Some concerns | Low risk | No concerns | No concerns | No concerns | No concerns | Moderate |
|  | tDCS:tDCS + ACUP | Some concerns | Low risk | No concerns | No concerns | Major concerns | Major concerns | Very low |
|  | ACUP:CRT | Some concerns | Low risk | No concerns | Some concerns | Major concerns | No concerns | Very low |
|  | ACUP:TMS | Some concerns | Low risk | No concerns | Major concerns | No concerns | No concerns | Low |
|  | ACUP:tDCS | Some concerns | Low risk | No concerns | Major concerns | No concerns | No concerns | Low |
|  | CRT:TMS | Some concerns | Low risk | No concerns | Major concerns | No concerns | No concerns | Low |
|  | CRT:tDCS | Some concerns | Low risk | No concerns | Some concerns | Major concerns | No concerns | Very low |
| FMA-LE | ACUP:TMS + ACUP | Some concerns | Low risk | No concerns | No concerns | No concerns | No concerns | Moderate |
|  | TMS:TMS + ACUP | Some concerns | Low risk | No concerns | No concerns | No concerns | No concerns | Moderate |
|  | CRT:TMS + ACUP | Some concerns | Low risk | No concerns | No concerns | No concerns | No concerns | Moderate |
|  | ACUP:tDCS +ACUP | Some concerns | Low risk | No concerns | Major concerns | No concerns | No concerns | Low |
|  | CRT:tDCS + ACUP | Some concerns | Low risk | No concerns | No concerns | No concerns | No concerns | Moderate |
|  | tDCS:tDCS + ACUP | Some concerns | Low risk | No concerns | Major concerns | No concerns | Major concerns | Very low |
|  | ACUP:CRT | Some concerns | Low risk | No concerns | Some concerns | Major concerns | No concerns | Very low |
|  | ACUP:TMS | Some concerns | Low risk | No concerns | Major concerns | No concerns | No concerns | Low |
|  | ACUP:tDCS | Some concerns | Low risk | No concerns | Major concerns | No concerns | No concerns | Low |
|  | CRT:TMS | Some concerns | Low risk | No concerns | Major concerns | No concerns | No concerns | Low |
|  | CRT:tDCS | Some concerns | Low risk | No concerns | Major concerns | No concerns | No concerns | Low |
| MAS | ACUP:TMS + ACUP | Some concerns | Low risk | No concerns | No concerns | No concerns | No concerns | Moderate |
|  | TMS:TMS + ACUP | Some concerns | Low risk | No concerns | No concerns | No concerns | No concerns | Moderate |
|  | ACUP:TMS | Some concerns | Low risk | No concerns | Some concerns | Some concerns | No concerns | Very low |
| BBS | ACUP:TMS + ACUP | Some concerns | Some concerns | No concerns | No concerns | No concerns | No concerns | Low |
|  | TMS:TMS + ACUP | Some concerns | Some concerns | No concerns | No concerns | No concerns | No concerns | Low |
|  | ACUP:tDCS + ACUP | Some concerns | Some concerns | No concerns | No concerns | No concerns | No concerns | Low |
|  | CRT:tDCS + ACUP | Some concerns | Some concerns | No concerns | No concerns | No concerns | No concerns | Low |
|  | ACUP:CRT | Some concerns | Some concerns | No concerns | No concerns | No concerns | No concerns | Low |
|  | ACUP:TMS | Some concerns | Some concerns | No concerns | Some concerns | No concerns | No concerns | Very low |
| MBI | ACUP:TMS + ACUP | Some concerns | Low risk | No concerns | No concerns | No concerns | No concerns | Moderate |
|  | TMS:TMS + ACUP | Some concerns | Low risk | No concerns | No concerns | No concerns | No concerns | Moderate |
|  | CRT:TMS + ACUP | Some concerns | Low risk | No concerns | No concerns | No concerns | Some concerns | Low |
|  | ACUP:tDCS + ACUP | Some concerns | Low risk | No concerns | No concerns | No concerns | Some concerns | Low |
|  | CRT:tDCS + ACUP | Some concerns | Low risk | No concerns | No concerns | No concerns | No concerns | Moderate |
|  | tDCS:tDCS + ACUP | Some concerns | Low risk | No concerns | No concerns | No concerns | Major concerns | Low |
|  | ACUP:CRT | Some concerns | Low risk | No concerns | No concerns | Some concerns | Major concerns | Very low |
|  | ACUP:TMS | Some concerns | Low risk | No concerns | Some concerns | No concerns | No concerns | Low |
|  | ACUP:tDCS | Some concerns | Low risk | No concerns | Major concerns | No concerns | Major concerns | Very low |
|  | CRT:TMS | Some concerns | Low risk | No concerns | No concerns | Some concerns | No concerns | Low |

**Supplementary Table S6**. Basic features of the included studies.

|  | **Reference** | **Sample size (M/F)** | **Age (Mean±SD)** | **Type of stroke (I/H)** | **Course of disease d/wk/m (Mean±SD)** | **Intervention** | **Treatment course** | **TMS** | **Outcome measures** |
| --- | --- | --- | --- | --- | --- | --- | --- | --- | --- |
| 1 | Zhu 2017 | I: 20 (11/9) C1: 20 (9/11) C2: 20 (10/10) | I: 64.23±10.54 C1: 61.00±11.68 C2: 65.45±13.12 | I: 10/10 C1: 9/11 C2: 11/9 | I: 34.45±11.61 d C1: 37.20±14.74 d C2: 35.68±12.73 d | I: rTMS + Acupuncture (scalp+body) C1: rTMS C2: Acupuncture | 5×/wk, 4wk | LF-rTMS(Magstim RAPID2): 1 Hz, Contralateral M1 | FMA,  WMFT |
| 2 | Zhang 2018 | I: 50 (34/16) C: 50 (33/17) | I: 55.9±7.5 C: 56.4±7.1 | I: 33/17 C: 34/16 | I: 2.7±0.7 m C: 2.6±0.9 m | I: rTMS + Acupuncture (Xing Nao Kai Qiao) C: rTMS | 5×/wk, 10wk | HF-TMS(MagProR100): 5 Hz, Left DLPFC | FMA, FAC, 10mWT, TUGT, BI |
| 3 | Zhao 2018 | I: 8 (7/1) C: 9 (7/2) | I: 53.75±12.33 C: 50.44±13.06 | I: 8/0 C: 9/0 | 2wk – 6 m | I: rTMS + Scalp Acupuncture C: Scalp Acupuncture | 14d | LF-rTMS(Magstim RAPID2): 1 Hz, Contralateral M1 | FMA,  MBI |
| 4 | Ma 2019 | I: 60 (38/22) C: 60 (43/17) | I: 56.17±11.24 C: 57.33±12.83 | I: 60/0 C: 60/0 | I: 59.43±8.16h C: 58.67±11.43h | I: tDCS + Scalp Acupuncture C: tDCS | 5×/wk, 4wk | tDCS(Sichuan ZCJM IS200): UE: Anode, C3 /C4, Cathode: Contralateral prefrontal cortex. LE: Anode, Cz, Cathode: Contralateral supraorbital region | ADL,  BI,  FMA |
| 5 | Sun 2019 | I: 40 (28/12) C1: 40 (27/13) C2: 40 (25/15) C3: 40 (25/15) | I: 65.83±5.10 C1: 65.80±4.98 C2: 65.92±5.12 C3: 65.76±4.82 | NR | I: 27.86±4.32d C1: 27.90±4.30d C2: 27.93±4.29d C3: 27.85±4.28d | I: rTMS + Scalp Acupuncture C1: rTMS C2: Scalp Acupuncture (Interactive) C3: CRT | 3m | LF-TMS(Magstim Rapid2): 1 Hz, Contralateral M1 | FMA-UE, EMS,  MBI |
| 6 | Cai 2020 | I: 40 (29/11) C1: 40 (26/14) C2: 40 (25/15) | I: 60.9±3.1 C1: 61.1±3.3 C2: 60.3±3.3 | I: 31/9 C1: 27/13 C2: 28/12 | I: 70.4±8.0d C1: 71.2±8.1d C2: 70.1±8.2d | I: rTMS + Scalp Electroacupuncture C1: rTMS C2: Scalp Electroacupuncture | 5×/wk, 2wk | LF-rTMS (YRD CYY-I): 1 Hz, Contralateral M1 of UE | FMA-UE,  BI,  MI-UL |
| 7 | Huang 2020 | I: 22 (15/7) C1: 22 (16/6) C2: 22 (14/8) | I: 55.73±11.83 C1: 55.25±11.73 C2: 56.14±10.24 | I: 15/7 C1: 15/7 C2: 17/5 | I: 64.10±16.89d C1: 63.77±18.42d C2: 62.81±16.74d | I: rTMS + Acupuncture C1: Acupuncture (luan san zhen) C2: rTMS | 5×/wk,  4wk | LF-rTMS (Magneuro100): 1 Hz, Contralateral M1 of UE | FMA-UE,  MBI |
| 8 | Kim 2020 | I: 11 (4/7) C1: 8 (5/3) C2: 11 (7/4) C3: 12 (7/5) | I: 67.55±12.53 C1: 67.00±12.92 C2: 64.45±14.75 C3: 62.17±16.25 | I: 11/0 C1: 8/0 C2: 11/0 C3: 12/0 | <1 m | I: rTMS + Scalp acupuncture C1: Scalp acupuncture C2: rTMS C3: CRT | Baseline: 5×/wk, 3wk follow up: 4wk | LF-rTMS(Magstim): 1 Hz, Contralateral M1 | FMA,  MBI,  FIM,  9HPT |
| 9 | Cui 2021 | I: 40 (28/12) C1: 40 (28/12) C2: 40 (27/13) | I: 64.87±5.59 C1: 64.87±5.59 C2: 65.27±5.14 | I: 40/0 C1: 40/0 C2: 40/0 | I: 6.47±1.20w C1: 6.27±1.32w C2: 6.57±1.20w | I: tDCS + Eye Acupuncture - Movement Therapy C1:Eye Acupuncture-Movement Therapy C2: CRT | 5×/wk, 4wk | tDCS(Jiangsu RHKL): Anode, Ipsilesional M1. Cathode, Contralateral Shoulder | FMA,  BI,  BBS |
| 10 | Ji 2021 | I: 30 (17/13) C: 30 (16/14) | I: 55.5±3.7 C: 55.3±3.2 | I: 14/16 C: 15/15 | I: 2.9±0.2m C: 2.7±0.3m | I: rTMS +Scalp Acupuncture (Scalp Penetrating Acupuncture) C: rTMS | 5×/wk, 3wk | LF-rTMS (YRD CCY-I): 1 Hz, Contralateral M1 of UE | FMA-UE, FMA-LE, FMA-B, MBI |
| 11 | Wang 2021 | I: 19 (11/8) C1: 19 (11/8) C2: 20 (8/12) | I: 60.95±9.31 C1: 63.95±6.79 C2: 58.95±7.20 | NR | I: 1.20±0.66m C1: 1.27±0.76m C2: 1.22±0.74m | I: tDCS + Acupuncture C1: Acupuncture C2: tDCS | 5×/wk, 4wk | tDCS(Sichuan ZCJM IS200): Anode : Ipsilesional M1. Cathode: Contralateral prefrontal cortex | FMA-UE, WMFT, MBI, Brunnstrom-UE,Brunnstrom-H |
| 12 | Yang 2021 | I: 51 (30/21) C: 51 (32/19) | I: 64.62±6.54 C: 65.36±6.44 | I: 23/28 C: 24/27 | I: 40.21±5.44d C: 41.42±5.60d | I: rTMS + Scalp Acupuncture C: Scalp Acupuncture | 7×/wk, 8wk | LF-rTMS (MagstimRapid2): 1 Hz, Baihui (GV20) and Ipsilesional Zhengying (GB17) | FMA-UE,  BI,  MAS |
| 13 | Dan 2022 | I: 30 (20/10) C1: 30 (25/5) C2: 30 (22/8) | I: 59.25±10.89 C1: 60.15±10.42 C2: 59.25±10.96 | I: 30/0 C1: 30/0 C2: 30/0 | I: 3.02±0.61m C1: 2.72±0.49m C2: 2.79±0.53m | I: rTMS + Acupuncture C1: rTMS + sham Acupuncture C2: sham rTMS + Acupuncture(Scalp cluster needling, scalp+body) | 6×/wk, 4wk | LF-rTMS (Magstim 200): 1 Hz, Contralateral cerebellar | FMA-B,  BBS |
| 14 | Deng 2022 | I: 48 (37/11) C: 48 (34/14) | I: 67.26±1.84 C: 67.18±1.79 | NR | NR | I: rTMS + Acupuncture (Xing Nao Kai Qiao) C: rTMS | 5×/wk, 4wk | LF-rTMS (DK-IIIB): 1 Hz, Contralateral M1 | FMA-UE, MBI, BBS |
| 15 | Li 2022 | I: 47 (29/18) C: 47 (32/15) | I: 65.88±12.51 C: 66.07±11.56 | I: 16/31 C: 18/29 | <3 m | I: rTMS + Acupuncture C: Acupuncture(Xing Shen Kai Qiao) | 5×/wk, 4wk | HF-rTMS (MagVenture): 10 Hz, Left DLPFC | FMA,BBS,MBI,ARAT,SS-QOL |
| 16 | Cao 2023 | I: 51 (27/24) C: 51 (30/21) | I: 60.13±6.31 C: 59.84±6.25 | I: 51/0 C: 51/0 | I: 19.02±3.12d C: 18.23±3.05d | I: rTMS + Acupuncture (Twelve Hand - Foot points) C: rTMS | LF-rTMS : 5×/wk, 4wk Acupuncture:3×/wk, 4wk | LF-rTMS (YRD CCY-I): 1 Hz, Contralateral M1 | FMA,  BBS,  MMT |
| 17 | Chen 2023 | I: 30 (18/12) C: 31 (16/15) | I: 65.80±6.12 C: 65.30±7.02 | I: 20/10 C: 23/8 | I: 63.17±18.33d C: 63.58±19.42d | I: rTMS + iTBS + Electroacupuncture C: Electroacupuncture(scalp + body) | 5×/wk, 4wk | LF-rTMS & iTBS(YRD CCY-I): LF-rTMS: 1Hz, Contralateral M1. iTBS: 50 Hz, Ipsilesional M1 | FMA-UE, MBI,  MAS |
| 18 | Cheng 2023 | I: 41 (22/19) C1: 42 (24/18) C2: 41 (23/18) C3: 40 (23/17) | I: 62.86±7.63 C1: 62.35±7.18 C2: 63.10±7.95 C3: 62.57±7.14 | I: 30/11 C1: 30/12 C2: 29/12 C3:31/9 | NR | I: tDCS + Acupuncture C1: tDCS C2: Acupuncture (scalp + body) C3: CRT | 7×/wk,  3 m | tDCS(STIMULATOR MC): Anode: Ipsilesional M1 of UE Cathode: Contralateral M1 of UE | FMA-UE, FMA-LE |
| 19 | Dang 2023 | I: 50 (30/20) C: 50 (28/22) | I: 71.89±7.62 C: 72.42±8.14 | I: 50/0 C: 50/0 | I: 3.46±0.75w C: 3.62±0.82w | I: rTMS + Acupuncture (Qi-Invigorating and Blood-Activating, scalp + body） C: rTMS | 6×/wk, 4wk | HF-rTMS (YRD CCY-IA): 5 Hz, Contralateral DLPFC | FMA-UE, FMA-LE, FMA,  ADL |
| 20 | Huang 2023 | I: 40 (20/20) C1: 40 (22/18) C2: 40 (25/15) | I: 61.47±6.74 C1: 60.23±5.43 C2: 59.35±6.33 | I: 28/12 C1: 30/10 C2: 32/8 | I: 45.39±7.96d C1: 46.74±8.32d C2: 44.25±9.10d | I: rTMS + Acupuncture C1: Acupuncture C2: rTMS | LF-rTMS : 5×/wk, 4wk Acupuncture:6×/wk, 4wk | LF-rTMS (Magneuro100): 1 Hz, Contralateral M1 | FMA-LE,  BI,  MMT,  MAS |
| 21 | Liu 2023a | I: 50 (28/22) C: 50 (30/20) | I: 73.05±6.31 C: 72.37±5.63 | NR | I: 21.41±5.61d C: 20.21±5.44d | I: rTMS + Electroacupuncture C: Electroacupuncture(scalp + body) | LF-rTMS:5×/wk, 4wk; Electroacupuncture: 6×/wk, 4wk | LF-rTMS(YRD CCY-I): 1 Hz, Contralateral M1 | FMA-UE, MAS |
| 22 | Liu 2023b | I: 60 (36/24) C: 60 (35/25) | I: 57.83±5.64 C: 58.42±6.33 | I: 60/0 C: 60/0 | I: 6.04±2.15m C: 6.13±1.87m | I: rTMS + Acupuncture (Warm Needling, body) C: rTMS | 5×/wk,3m | LF-rTMS (YRD MagTD): 1 Hz, Contralateral M1 | FMA-UE, FMA-LE, SS-QOL |
| 23 | Meng 2023 | I: 39 (26/13) C: 39 (25/14) | I: 66.31±5.73 C: 67.00±5.99 | I: 24/15 C: 26/13 | I: 26.18±2.01d C: 25.49±2.91d | I:rTMS+Electroacupuncture(scalp+body) C: rTMS | 5×/wk, 4wk | LF-rTMS (YRD CCY-I): 1 Hz, Right DFL | FMA |
| 24 | Wang 2023a | I: 30 (20/10) C: 30 (18/12) | I: 57.18±12.06 C: 56.43±11.27 | I: 22/8 C: 20/10 | I: 67.5±8.76d C: 63.6±9.61d | I: rTMS + Acupuncture (electrothermal) C: rTMS | 5×/wk, 4wk | LF-rTMS: 1 Hz, Contralateral M1 | FMA-UE, MBI,  MAS |
| 25 | Wang 2023b | I: 40 (25/15) C1: 40 (22/18) C2: 40 (23/17) | I: 60.25±5.84 C1: 56.68±6.23 C2: 54.07±6.15 | I: 40/0 C1: 40/0 C2: 40/0 | I: 1.15±0.78m C1: 1.16±0.64m C2: 1.30±0.71m | I: tDCS + Acupuncture(body) C1: Acupuncture C2: tDCS | 5×/wk,  4wk | tDCS(Sichuan ZCJM IS200): Anode: Ipsilesional M1 Cathode: Contralateral prefrontal cortex | FMA-UE,  MBI |
| 26 | Wang 2023c | I: 35 (21/14) C: 35 (24/11) | I: 60.88± 8.28 C: 64.40± 10.20 | NR | I: 48.50±14.33d C: 45.63±12.26d | I: rTMS + Acupuncture C: acupuncture(scalp + body) | 6×/wk, 3wk | LF-rTMS(M-100Ultlmate): 1 Hz, Contralateral M1 | FMA-UE, MBI,  NHPT |
| 27 | Xie 2023 | I: 17 (10/7) C: 19 (13/6) | I: 54.47±9.152 C: 58.42±12.76 | I: 10/7 C: 14/5 | I: 21.33±13.04d C: 17.33±8.72d | I: rTMS + Acupuncture (Xing Nao Kai Qiao) C: Acupuncture (Xing Nao Kai Qiao) | 5×/wk, 2wk | LF-rTMS (YRD CCY-I): 1 Hz, Contralateral M1 | FMA-UE, MBI, MAS, Brunnstrom-UE  Brunnstrom-H |
| 28 | Yu 2023 | I: 35 (23/12) C: 35 (24/11) | I: 55.94±13.32 C: 52.94±12.35 | I: 5/30 C: 4/31 | I: 6.55±2.64w C: 6.47±2.72w | I: rTMS + Acupuncture + CRT C: Acupuncture + CRT | 5×/wk, 6wk | LF-rTMS (YRD CCY-I): 1 Hz, Contralateral M1 | FMA, BI |
| 29 | Zhang 2023 | I: 60 (34/26) C: 60 (32/28) | I: 63.8± 10.0 C: 61.9±12.3 | I: 34/26 C: 30/30 | I: 2.57±0.81m C: 2.37±0.58m | I: tDCS + Acupuncture(scalp + body) C: tDCS | 5×/wk, 3wk | tDCS(Sichuan ZCJM IS200): Anode, Right IPL, Ipsilesional STG and Prefrontal lobe; Cathode, Contralateral prefrontal lobe | FMA, CBIT-HK, MBI, FIM |
| 30 | Zhou 2023 | I: 30 (26/4) C: 30 (22/8) | I: 55.67±10.36 C: 60.23±8.87 | NR | I: 8.43±5.82w C: 10.13±6.49w | I: HF&rTMS + acupuncture(scalp +body) C: acupuncture(body + sham scalp) | 5×/wk, 2wk | LF&HF rTMS(YRD): 5 Hz, Ipsilesional M1(Morning); 1 Hz, Contralateral M1(Afternoon) | FMA-UE, BI, FTHUE-HK |
| 31 | Chen 2024 | I: 34 (20/14) C1: 37 (25/12) C2: 35 (23/12) | I: 58.74±7.45 C1: 57.12±7.24 C2: 57.45±7.36 | I: 26/8 C1: 24/13 C2: 25/10 | I: 58.62±8.14 d C1: 55.74±8.32 d C2: 58.15±7.65 d | I: rTMS + Stage Acupuncture C1: LF‑rTMS C2: Stage Acupuncture | 6×/wk, 6wk | LF-TMS(YRD CCY): 1 Hz, Contralateral M1 | FMA-UE, MBI, MAS, Brunnstrom |
| 32 | Pu 2024 | I: 30 (16/14) C1: 30 (18/12) C2: 30 (19/11) | I: 55.13±6.59 C1: 60.80±7.34 C2: 60.66±7.38 | NR | I: 41.20±27.17 d C1: 29.93±4.54 d C2: 29.53±4.62 d | I: rTMS + Stage Acupuncture-Movement Therapy C1:Stage Acupuncture-Movement Therapy C2: Stage Acupuncture | 5×/wk, 4wk | LF-TMS(Magneuro10): 1 Hz, Contralateral M1 | FMA‑UE, MBI, STEF |
| 33 | Wang 2024 | I: 38 (21/17) C: 38 (18/20) | I: 54±10 C: 55±10 | I: 19/19 C: 22/16 | I: 2.01±0.43m C: 1.87±0.36m | I: rTMS + Acupuncture (moxibustion and acupuncture of the governor vessel, scalp + body) C: rTMS | 14d | LF-rTMS(M-100 Ultimate): 1 Hz, Contralateral M1 & DLPFC | FMA |
| 34 | Xiao 2024 | I: 49 (28/21) C: 49 (25/24) | I: 67.38±6.93 C: 69.03±6.84 | I: 49/0 C: 49/0 | I: 8.20±2.01w C: 8.11±2.05w | I: rTMS + Acupuncture(Kai Qiao Xing Shen Buxu Tongluo) C: rTMS | 5×/wk, 3m | LF-TMS(Dalian THL-120): 1 Hz, Contralateral M1 | FMA,  BBS,  6MWD |
| 35 | Xu 2024 | I: 29 (15/14) C: 28 (12/16) | I: 56.69 ±1 4.68 C: 60.64±12.24 | I: 30/0 C: 30/0 | I: 7.17±2.74d C: 7.25±2.76d | I: tDCS + Acupuncture C: tDCS | 2×/d,  14 d | tDCS (DK-501): Anode, Ipsilesional M1; Cathode, contralateral shoulder | FMA‑H, MMT, JTT, Brunnstrom, |
| 36 | Zhang 2024 | I: 30 (19/11) C: 30 (21/9) | I: 59.77±7.79 C: 55.53±9.53 | I: 30/0 C: 30/0 | I: 16.57±5.72d C: 17.70±5.87d | I: rTMS + Scalp Acupuncture C: CRT | 5×/wk, 4wk | LF-TMS(Magneuro100): 1 Hz, Contralateral M1 | FMA‑LE, MBI, FAC |
| 37 | Zhao 2024 | I: 30 (20/10) C: 30 (19/11) | I: 63.4±13.3 C: 65.2±8.5 | I: 30/0 C: 30/0 | I:6.8±2.1d C:7.0±2.1d | I: iTBS + Electroacupuncture C: iTBS | 5×/wk, 3m | iTBS: 50 Hz, Contralateral cerebellar | FMA, MBI |
| 38 | Zhu 2024 | I: 30 (22/8) C: 30 (23/7) | I: 55.8±12.7 C: 52.5±10.3 | I: 21/9 C: 17/13 | I: 55.7±38.4d C: 60.1±36.7d | I: rTMS +Acupuncture (Guan Ci, body) C: rTMS | 5×/wk, 3wk | LF-TMS(Shenzhen Ultimate): 1 Hz, Contralateral M1 | FMA-UE, MBI, MAS |
| 39 | Gao 2025 | I: 58 (24/34) C: 58 (32/26) | I: 69.88±6.43 C: 70.73±6.98 | I: 28/30 C: 23/35 | I: 6.33±1.42m C: 6.20±1.65m | I: HF- rTMS + Acupuncture (Jin’s triple- needle therapy, scalp +body) C: LF & HF rTMS | 5×/wk, 4wk | HF rTMS(Tenda TC100): 10 Hz, Ipsilesional & Contralateral M1 | FMA-UE, FMA-LE, FMA, SS-QOL |
| 40 | Wang 2025a | I: 51 (34/17) C: 51 (31/20) | I: 62.18±7.06 C: 63.63±7.48 | I: 51/0 C: 51/0 | I: 3.98±0.55 m C: 4.06±0.51 m | I: HF-rTMS + Electroacupuncture C: HF-rTMS | 5×/wk, 8wk | HF-rTMS(YRD CCY-1): 10 Hz, ipsilesional DLPFC | FMA-UE,  FMA-LE, MBI,MAS, NIHSS |
| 41 | Wang 2025b | I: 45 (24/21) C: 45 (23/22) | I: 63.12±4.91 C: 63.38±4.84 | NR | NR | I: CRT + rTMS + Acupuncture (Tongdu Tiaoshen)  C: CRT | 5×/wk, 8wk | HF-rTMS(NK-IB02): 10 Hz, left DLPFC | FMA,BI,  NIHSS, SS-QOL |
| 42 | Yang 2025 | I: 49 (33/16) C: 49 (31/18) | I: 50.73±4.45 C: 51.39±4.50 | I: 49/0 C: 49/0 | I: 4.78±1.32 wk C: 4.82±1.35 wk | I: rTMS + Wrist-ankle acupuncture + CRT C: CRT | 10 d | HF-rTMS(Magstim Rapid2): 10 Hz, ipsilesional M1 | FMA-UE, FMA-LE, NIHSS,BBS |
| 43 | Su 2026 | I: 41 (19/22) C: 41 (18/23) | I: 66.28±4.09 C: 66.23±4.12 | NR | I: 3.99±0.42 m C: 3.95±0.45 m | I: rTMS + Acupuncture(Tongtiao Du Meridian) + cognitive rehabilitation C: rTMS + cognitive rehabilitation | 5×/wk, 4wk | HF-rTMS(HX-C3): 10 Hz, target NR | FMA, MBI |
| 44 | Wang 2026 | I: 80 (48/32) C: 80 (45/35) | I: 61.64±6.84 C: 60.58±6.49 | I: 80/0 C: 80/0 | I: 38.35±5.58 d C: 36.73±6.17 d | I: rTMS + Acupuncture + CRT C: rTMS + CRT | 6×/wk, 8wk | LF-rTMS(NK-IC04): 1–5 Hz, contralateral M1 | FMA, BBS, FAC, BI |
| 45 | Xue 2026 | I: 38 (20/18) C: 38 (24/14) | I: 62.71±5.40 C: 62.09±3.18 | I: 38/0 C: 38/0 | I: 4.34±0.80 wk C: 4.29±0.84 wk | I: rTMS + Acupuncture ( Xingnao Kaiqiao) +CRT C: CRT | 5×/wk, 8wk | LF-rTMS: 1 Hz, contralateral M1 | FMA,MBI,  NIHSS, SS-QOL, |
| **Notes: Values are reported as the means ± SD unless otherwise stated. M/F indicates male/female. I/H indicates ischemic/hemorrhagic stroke. d/wk/m indicates days/weeks/months. I denotes the intervention group, and C (C1/C2/C3) denotes the control group(s) in multiarm trials. Abbreviations: 10mWT, 10-Meter Walk Test. 6MWD, six-minute walk distance. ADL, Activities of Daily Living. ARAT, Action Research Arm Test. BBS, Berg Balance Bcale. BI, Barthel Index. Brunnstrom-H, Brunnstrom for Hand. Brunnstrom-UE, Brunnstrom for Upper Extremity. CBIT-HK, Comprehensive Behavioral Intervention for Tics, Hong Kong Version. CRT, conventional rehabilitation therapy. DFL, Dorsolateral frontal lobe. DLPFC, dorsolateral prefrontal cortex. EMS, Elderly Mobility Scale. FAC, Functional Ambulation Category. FIM, Functional Independence Measure. FMA, Fugl-Meyer Assessment. FMA-B, Fugl-Meyer Assessment for Balance. FMA-LE, Fugl-Meyer Assessment for Lower Extremity. FMA-UE, Fugl-Meyer Assessment for Upper Extremity. FTHUE-HK, functional test of the hemiplegic upper extremity, Hong Kong version. iTBS, intermittent theta-burst stimulation. IPL, inferior parietal lobule. JTT, Jebsen-Taylor hand function test. M1, primary motor cortex. MAS, modified Ashworth scale. MBI, Modified Barthel Index. MMT, Manual Muscle Testing. MI-UL, upper limb motility index. NHPT (also reported as 9HPT), Nine-Hole Peg Test. NR, not reported. rTMS, repetitive transcranial magnetic stimulation. tDCS, transcranial direct current stimulation. SS-QOL, Stroke-Specific Quality of Life Scale. STEF, Simple Test for Evaluating Hand Function. STG, superior temporal gyrus. TMS, transcranial magnetic stimulation. TUGT, Timed Up and Go Test. UE, upper extremity. LE, lower extremity. WMFT, Wolf Motor Function Test. HF, high frequency. LF, low frequency.** | | | | | | | | | |
